# SUMOylation by the E3 Ligase TbSIZ1/PIAS1 Positively Regulates VSG Expression in *Trypanosoma brucei*

**DOI:** 10.1371/journal.ppat.1004545

**Published:** 2014-12-04

**Authors:** Diana López-Farfán, Jean-Mathieu Bart, Domingo I. Rojas-Barros, Miguel Navarro

**Affiliations:** 1 Instituto de Parasitología y Biomedicina “López-Neyra”, Consejo Superior de Investigaciones Científicas, CSIC (Spanish National Research Council), Parque Tecnológico de Ciencias de la Salud, Granada, Spain; 2 Centro Nacional de Medicina Tropical, Instituto de Salud Carlos III (ISCIII), Madrid, Spain; University of California, Los Angeles, United States of America

## Abstract

Bloodstream form trypanosomes avoid the host immune response by switching the expression of their surface proteins between Variant Surface Glycoproteins (VSG), only one of which is expressed at any given time. Monoallelic transcription of the telomeric *VSG* Expression Site (ES) by RNA polymerase I (RNA pol I) localizes to a unique nuclear body named the ESB. Most work has focused on silencing mechanisms of inactive *VSG*-ESs, but the mechanisms involved in transcriptional activation of a single *VSG*-ES remain largely unknown. Here, we identify a highly SUMOylated focus (HSF) in the nucleus of the bloodstream form that partially colocalizes with the ESB and the active *VSG*-ES locus. SUMOylation of chromatin-associated proteins was enriched along the active *VSG*-ES transcriptional unit, in contrast to silent *VSG*-ES or rDNA, suggesting that it is a distinct feature of *VSG*-ES monoallelic expression. In addition, sequences upstream of the active *VSG*-ES promoter were highly enriched in SUMOylated proteins. We identified TbSIZ1/PIAS1 as the SUMO E3 ligase responsible for SUMOylation in the active *VSG*-ES chromatin. Reduction of SUMO-conjugated proteins by TbSIZ1 knockdown decreased the recruitment of RNA pol I to the *VSG*-ES and the *VSG*-ES-derived transcripts. Furthermore, cells depleted of SUMO conjugated proteins by TbUBC9 and TbSUMO knockdown confirmed the positive function of SUMO for *VSG*-ES expression. In addition, the largest subunit of RNA pol I TbRPA1 was SUMOylated in a TbSIZ-dependent manner. Our results show a positive mechanism associated with active *VSG*-ES expression via post-translational modification, and indicate that chromatin SUMOylation plays an important role in the regulation of *VSG*-ES. Thus, protein SUMOylation is linked to active gene expression in this protozoan parasite that diverged early in evolution.

## Author Summary

African trypanosomes have evolved one of the most complex strategies of immune evasion by routinely switching the expression of surface proteins called Variant Surface Glycoproteins (VSG), only one of which is expressed at any given time. Previous work has suggested that the recruitment of a single *VSG* telomeric locus to a discrete nuclear body (ESB) underlies the mechanism responsible for *VSG* monoallelic expression. Our findings establish unexpected roles for SUMOylation as a specific post-translational modification that marks the ESB and the *VSG*-ES chromatin. We describe a highly SUMOylated focus (HSF) as a novel nuclear structure that partially colocalizes with the VSG-ES locus and the nuclear body ESB. Furthermore, chromatin SUMOylation is a distinct feature of the active *VSG*-ES locus, in contrast to other loci investigated. SUMOylation of chromatin-associated proteins is required for efficient recruitment of the polymerase to the VSG-ES promoter and for *VSG*-ES expression. Altogether, these data suggest the presence of a large number of SUMOylated proteins associated with monoallelic expression as Protein Group SUMOylation. In contrast to the wealth of literature focused on *VSG* regulation by silencing, our results indicate a positive mechanism via SUMOylation to regulate VSG expression in the infectious form of this protozoan parasite.

## Introduction

*Trypanosoma brucei* displays a sophisticated mechanism of antigenic variation of the Variant Surface Glycoprotein (VSG) that allows the parasite to elude the host immune antibody response, ensuring a persistent infection [1], [2]. Antigenic variation is achieved by mutually exclusive expression of only one out of approximately 1000 *VSG* genes. The monoallelic expressed *VSG* gene is located at the end of a telomeric Expression Site (ES) locus. There are about 15 different *VSG* expression sites (*VSG*-ESs), which share highly homologous sequences at the promoter region [3]. The identification of a single extra-nucleolar RNA polymerase I-containing nuclear body, named the expression site body (ESB), which is associated with the GFP-tagged active *VSG*-ES promoter suggests a model whereby ESB-dependent *VSG*-ES recruitment leads to the expression of a single VSG on the surface of the parasite [4]–[6].

Transcription of the *VSG*-ES and maintaining monoallelic expression seem be controlled at multiple levels. Several proteins have been involved in silencing of inactive *VSG*-ESs, such as telomeric protein RAP1, DOT1 histone methyltransferase, the factor ISWI and chromatin remodeler complex FACT [7]–[10]. Recently, it has been reported that the active *VSG*-ES promoter is depleted of histones [11], [12]. Whilst most studies have focused on regulation of *VSG-*ES silencing, there must be specific factors required to guarantee high levels of transcription of the active *VSG*-ES. The architectural protein TDP1, a high mobility group (HMG) containing protein, facilitates RNA pol I activity, however is required for both *VSG*-ES and rDNA transcription [13].

In *T. brucei*, the *VSG*-ES is transcribed by RNA polymerase I (RNA pol I), an exceptional feature among eukaryotes since RNA pol I does not usually transcribe protein-coding genes. However, TbRPB7, a dissociable subunit of the RNA pol II complex, is also required for *in vivo* RNA pol I transcription of the *VSG* gene [14]. This is a controversial issue in the field since TbRPB7 does not seem to be required for *in vitro* transcription [15]. These discrepancies maybe explained by a possible function of TbRPB7 *in vivo*, as we discussed previously [16]. Based in our previous results we sought for TbRPB7-interacting proteins in search for possible factors involved in *VSG*-ES regulation. To do so, we directed a yeast two-hybrid screen (Hybrigenics) and identified several proteins, including a protein with a conserved SUMO E3 ligase domain (MIZ/SP-RING), that we named TbSIZ1.

SUMO (Small Ubiquitin-like MOdifier) is a reversible post-translational protein modification involved in many cellular processes, including the regulation of nuclear bodies. The first SUMO gene was identified in *S. cerevisiae* (*SMT3*); the peptide was found covalently attached to the Ran GTPase-activating protein, modifying the localization of this protein in the cell [17], [18]. SUMO are ∼12 kDa proteins with a 3D structure similar to ubiquitin, whilst sharing just 20% sequence identity. Invertebrates such as yeast, *C. elegans*, and *D. melanogaster* contain a single *SUMO* gene, whereas plants and vertebrates have several *SUMO* genes [19].

SUMOylation, like ubiquitylation, involves a pathway that requires three enzymatic steps. First, the SUMO protein is activated at its C terminus by the E1 activating enzyme [20]. The activated SUMO is then transferred to the E2 conjugating enzyme UBC9 and to the substrate forming an isopeptide bond. This last step is mediated by SUMO E3 ligases, which determine substrate specificity and catalyse the transfer of SUMO from UBC9 [21], [22].

Three protein families have been identified to date as SUMO E3 ligases. The main group is characterized by a conserved SP-RING motif, which is essential for their function. This group includes the PIAS family (Protein inhibitor of activated STAT) PIAS1-3 in mammals [23], and Siz1, Siz2 and Mms21 in budding yeast [21], [24]. One of their mechanisms consists in re-localization of transcriptional regulators to different subnuclear compartments [25]. The second type of SUMO E3 ligases is represented by the nuclear import factor RanBP2, which mediates nucleo-cytoplasmic transport [26]. The third group was discovered with the polycomb protein Pc2, which forms PcG nuclear bodies involved in gene silencing [27].

SUMO modification regulates protein activity in diverse ways. SUMO can modulate the ability of proteins to interact with their partners, alter their patterns of sub-cellular localization and control their stability. The most common group of SUMO substrates are transcription factors, whose transcriptional activity can be modulated positively or negatively as a result of SUMOylation [28].

In *T. brucei*, there is a single SUMO protein which has been shown to be essential in procyclic [29] and bloodstream forms [30] of the parasite. Recently, proteomic analysis of SUMO substrates in *T. cruzi* showed at least 236 proteins involved in several cellular processes [31]. Together these data suggest that SUMO is essential and SUMOylation is a conserved process in trypanosomatids. The lack of an anti-SUMO antibody specific for TbSUMO hampered a proper analysis of the SUMO conjugated proteins [32]. Thus, a possible SUMO function in gene expression and subcellular localization of SUMO-conjugated proteins in the infective form of this protozoan parasite are totally unknown.

We here show the presence of a single site in the nucleus highly enriched in SUMOylated proteins, which associates with the *VSG*-ES chromatin and the nuclear body ESB. Importantly, we identify the SUMO E3 ligase, named TbSIZ1, responsible for the *VSG*-ES chromatin SUMOylation. Our data indicate that SUMOylation of chromatin-associated proteins at the active *VSG*-ES promoter is highly enriched in a TbSIZ1-dependent manner. SUMOylation of chromatin-associated proteins contributes to efficient recruitment of RNA polymerase I to the *VSG*-ES promoter and is important for *VSG*-ES expression. In addition, RNA pol I largest subunit TbRPA1 is SUMOylated in a TbSIZ1-depending manner. However, additional chromatin-associated proteins are SUMOylated in the active *VSG*-ES since SUMO was detected upstream of the promoter. This epigenetic mark in chromatin was not detected in silent *VSG-*ESs nor in rDNA or EP transcribed also by RNA pol I, suggesting that SUMOylation is involved in *VSG*-ES monoallelic active expression rather than in silencing.

## Results

### Expression and localization of SUMOylated proteins in *Trypanosoma brucei*

**Figure 1:**
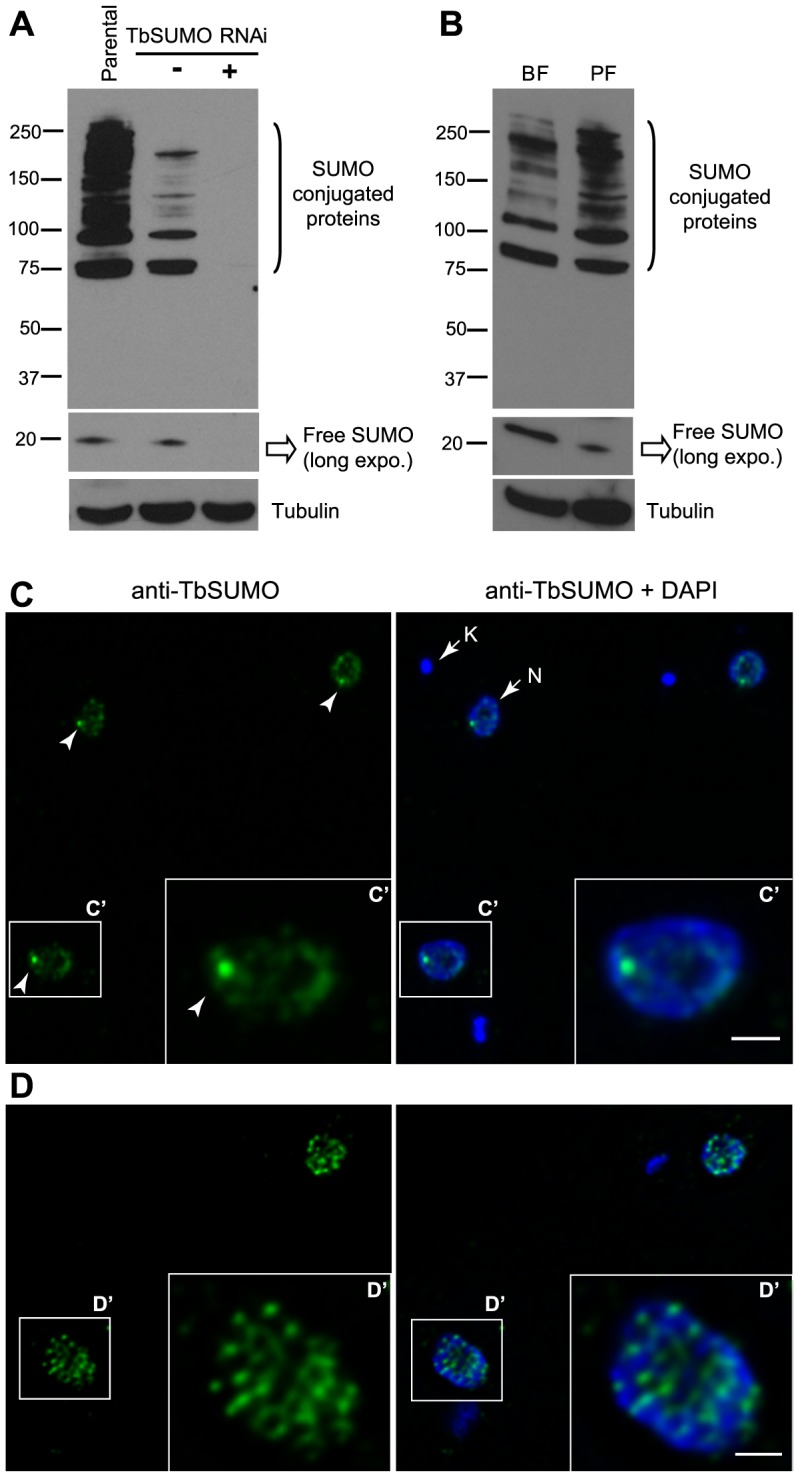
Expression pattern and subcellular localization of *T. brucei* SUMOylated proteins. (A) Multiple bands corresponding to SUMO conjugated proteins are reduced upon TbSUMO knockdown. Western blot analysis of SUMOylated proteins with a monoclonal antibody generated against TbSUMO (mAb 1C9H8). Whole cell extracts from bloodstream trypanosomes were prepared in the presence of 20 mM NEM and separated in 4–20% precast polyacrylamide gels (Bio-Rad). (5×10^6^ cells/lane); Parental, uninduced (−) and induced 48 h (+). Anti-tubulin was used as a loading control. (B) Expression pattern of SUMOylated proteins in two developmental forms of *T. brucei*. Bloodstream form (BF) and insect procyclic form (PF) total cell extracts were prepared in the presence of 20 mM NEM and analyzed by Western blot using the mAb 1C9H8. (C) SUMO conjugated proteins are diffusely distributed in the nucleoplasm including a Highly SUMOylated Focus (HSF) (arrowhead) in the bloodstream form of the parasite. Double indirect three-dimensional immunofluorescence (3D-IF) analysis was carried out using the anti-TbSUMO mAb 1C9H8 (green). (C′) Higher magnification of the nucleus showing anti-SUMO and DAPI fluorescence signals. DNA in the nucleus (N) and the kinetoplast (K) was stained with DAPI (blue). Statistical analysis showed the presence of a highly SUMOylated focus in 74.9% of the cells (Figure S2B). (D) SUMOylated proteins in the nucleus of procyclic form are diffusely distributed in many foci. 3D-IF analysis was carried out using the anti-TbSUMO mAb (green). (D′) Higher magnification of the nucleus showing anti-SUMO and DAPI fluorescence signals. DNA was stained with DAPI (blue). Scale bars 1 µm. Maximum intensity projections of deconvolved two-channel 3D stack are shown.

To investigate SUMO-conjugated protein expression we first developed a monoclonal antibody (mAb 1C9H8) against *Trypanosoma brucei* SUMO expressed as recombinant protein. Western blot analysis showed that the most abundant SUMO-conjugated proteins are larger than 70 kDa in bloodstream form trypanosome total extracts (Figure 1A), similar to the pattern described in other eukaryotes [21], [33]. The mAb 1C9H8 recognized free SUMO and SUMO-conjugated proteins since SUMO depletion by RNAi of the coding region showed a significant decreased signal after 48 h of depletion by Western blot analysis (Figure 1A). RNAi-induced lines were compared to the parental cell line since uninduced cell lines generally showed some depletion of the target protein due to leaky RNAi expression. Additional RNAi experiments using the TbSUMO 5′ UTR showed a similar depletion of SUMO by Western blot analysis (Figure S1A). Importantly, the use of N-Ethylmaleimide (NEM), a well-known inhibitor of de-sumoylases, reduced the signal of free SUMO in protein extracts and stabilized SUMO-conjugated proteins (Figure S1B), suggesting NEM inhibits trypanosome de-sumoylation. The previous use of the anti-*Trypanosoma cruzi* SUMO antiserum against *T. brucei* SUMO conjugated proteins [30] is controversial [32]. We compared the anti-TcSUMO rabbit antiserum on TbSUMO-depleted extracts by RNAi with the signal obtained using the anti-TbSUMO mAb on the same Western blot (Figure S1C). While the signal generated by the anti-TbSUMO mAb was abolished upon depletion, anti-TcSUMO signal was not significantly reduced. Altogether, these data suggest that the anti-TbSUMO mAb 1C9H8 showed specificity to recognize SUMO-conjugated proteins in *T. brucei* extracts. Comparative analysis of *T. brucei* total extracts in bloodstream and procyclic (insect form) developmental stages of the parasite showed differential expression pattern of several SUMO-conjugated proteins (Figure 1B).

Next, we analyzed the subcellular localization of SUMOylated proteins by three-dimensional immunofluorescence (3D-IF) microscopy using the mAb anti-TbSUMO 1C9H8. SUMO modified proteins localized mainly to the nucleus, excluding the nucleolus, in a diffuse pattern with one Highly SUMOylated Focus (HSF) (Figure 1C). Statistical IF analysis for the detection of this single HSF revealed a significant visualization in 74.9% of the nuclei, irrespective of cell cycle stage (Figures S2A and S2B). Conversely, in the procyclic insect form, where no VSG is expressed, SUMO-conjugated nuclear proteins are located in numerous small foci dispersed in the nucleus (Figure 1D).

### Nuclear SUMO-conjugated proteins are associated with the nuclear body ESB and the active *VSG*-ES

**Figure 2:**
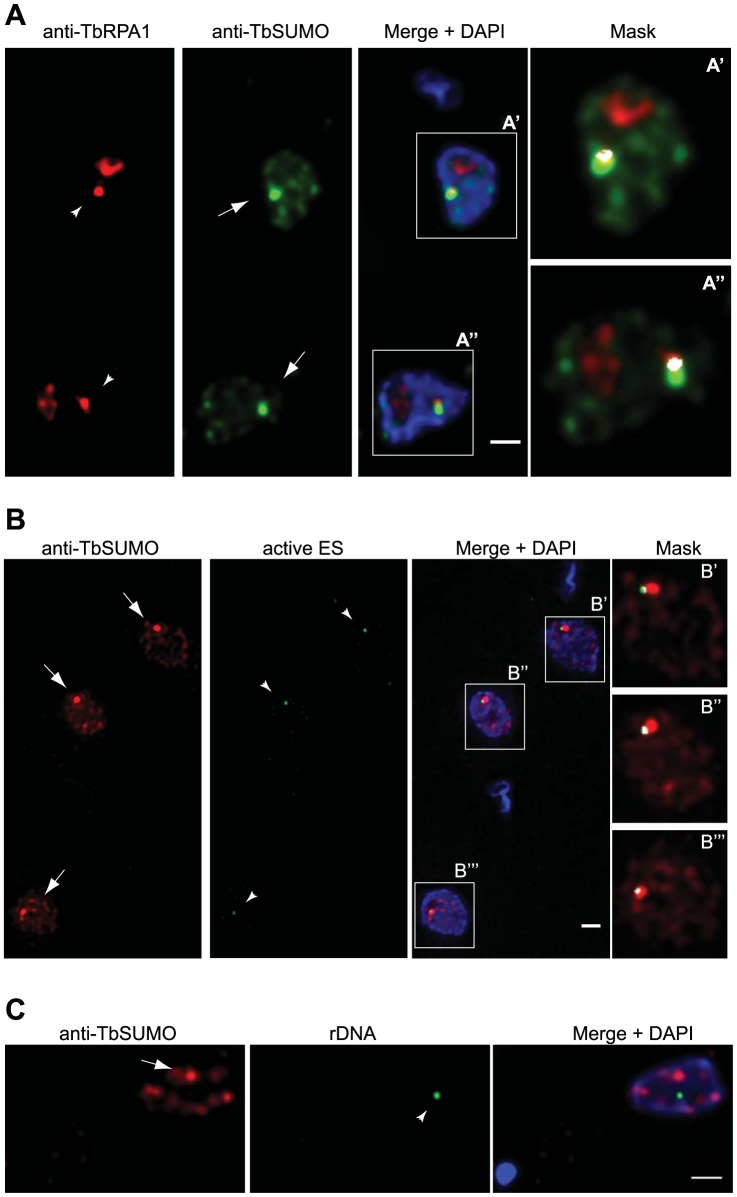
The Highly SUMOylated Focus (HSF) associates with the Expression Site Body (ESB) and the active *VSG*-ES. (A) SUMOylated proteins in the HSF partially colocalize with the RNA pol I in the ES. Double indirect 3D-IF analysis was performed in bloodstream form using anti-TbRPA1 antiserum (red), anti-TbSUMO mAb (green) and DAPI staining (blue). The ESB (arrowhead) and the Highly SUMOylated Focus (HSF) (arrow) are indicated. (A′, A″) Higher magnification of the nucleus showing anti-TbRPA1 and anti-TbSUMO fluorescence signals colocalization mask (white). The colocalization mask calculated for each nonequalized 8-byte slice and merged with both anti-TbRPA1 and anti-TbSUMO labelling, with a ratio setting value of 80%. Maximum intensity projections of deconvolved two-channel 3D stack are shown. See Figure S3 for HSF and ESB colocalization analysis throughout the cell cycle. (B) HSF associates with the active *VSG*-ES. Double indirect 3D-IF was performed in a cell line where the active *VSG*-ES was GFP-LacI tagged [4]. SUMO was detected with anti-TbSUMO mAb (red) and the GFP-tagged active ES with a rabbit anti-GFP antiserum (green). Maximum intensity projections of deconvolved slices containing the GFP dot signal are shown (arrowhead). (B′, B″, B′″) Higher magnification of the nucleus showing anti-GFP and anti-TbSUMO fluorescence signals colocalization mask (white), calculated as described above. See Figure S4 for *VSG*-ES and HSF colocalization analysis throughout the cell cycle. (C) SUMOylated nuclear proteins do not colocalize significantly with rDNA locus. Double 3D-IF analysis was performed in a cell line where the rDNA locus was GFP-tagged (arrowhead). SUMO was detected with anti-TbSUMO mAb (red) and the GFP-tagged active ES with a rabbit anti-GFP antiserum (green). Scale bars, 1 µm.

We carried out a series of double 3D-IF experiments to investigate a possible association of SUMO with trypanosome sub-nuclear compartments in bloodstream form nuclei. Anti-TbRPA1 (RNA pol I largest subunit) affinity-purified antiserum is known to recognize not only the nucleolus but also the extra-nucleolar body named ESB [4]. Double IF analysis by 3D-deconvolution microscopy using the mAb anti-TbSUMO and the anti-TbRPA1 antiserum showed that the HSF partially colocalized with the nuclear body ESB (Figure 2A). To further investigate the association between RNA pol I and SUMOylated proteins in the nucleus, 3D-IF analysis was performed in a cell line expressing a YFP-tagged TbRPB5z [34], a subunit specific of RNA pol I complex in trypanosomes. Consistent with the nuclear localization of TbRPA1, TbRPB5z was associated with the HSF in the nucleus (Figure S2C), suggesting that the RNA pol I complex located in the extra-nucleolar ESB is associated with the HSF.

Next, we wished to investigate whether the HSF was associated with the *VSG*-ES chromosome position in the nucleus. To do so, we performed indirect 3D-IF analysis utilizing a cell line tagged with the GFP-Lac upstream of the active *VSG*-ES promoter [4]. Double 3D-IF analysis using anti-TbSUMO and anti-GFP antibodies showed that the GFP-tagged active *VSG*-ES partially colocalized with the HSF in a large percentage of cells (76.1%) (Figures 2B and S2D). As control, we investigated a possible association of SUMOylated proteins with the ribosomal DNA (rDNA) in the nucleus, another locus transcribed by RNA pol I. Nuclear position analysis of the rDNA locus, marked with the GFP-Lac [35], showed a lack of significant colocalization with SUMOylated proteins (Figures 2C and S2D).

The monoallelic *VSG*-ES transcriptional state is maintained over many generations and during S-phase, G2-phase and early mitosis the active *VSG*-ES locus remains associated with the single ESB [34]. Thus, we decided to investigate the dynamic of the HSF throughout the cell cycle. Trypanosome cell cycle phases are clearly distinguishable because kinetoplast mitochondrial DNA (K) segregation occurs prior to the onset of mitosis and nuclear (N) division. Thus, DAPI staining identifies a population with 1K1N cells (G1 and G1-S) and 2K1N cells (G2). To analyze HSF dynamics throughout the cell cycle we performed double indirect 3D-IF in bloodstream form trypanosomes using antiserum against TbRPA1 and the mAb anti-TbSUMO. This analysis revealed that the HSF and the ESB partially colocalized in every stage of the cell cycle (Figure S3). Anti-TbSUMO labeling allowed us now to distinguish in the nucleus the ESB when is located closed of the nucelolus (Figure S3, G1 cell).

In pre-mitotic cells a single ESB remained associated to the *VSG*-ES sister chromatids during segregation [34]. Thus, we investigated a possible association of the HSF with the active *VSG*-ES chromatids during the cell cycle using the GFP-tagged *VSG*-ES cell line. Double 3D-IF analysis using anti-SUMO and anti-GFP antibodies showed a single HSF in the nucleus, which was associated with the active *VSG*-ES locus throughout the cell cycle. Interestingly, the two sister chromatids of the active *VSG*-ES in pre-mitotic cells were associated with a single HSF. Once cells enter into mitosis and sister chromatids are clearly separated, two HSFs associated with each chromatid were detected (Figure S4).

### SUMOylation of chromatin-associated proteins is a distinct feature of the active *VSG*-ES locus

Nuclear localization analysis by 3D-IF analysis suggested that SUMO-conjugated proteins associate with the active *VSG*-ES telomeric locus in the bloodstream form (Figure 2B). Next, we decided to investigate in detail the occupancy of SUMOylated proteins along the *VSG*-ES locus by chromatin immunoprecipitation (ChIP) analysis and quantitative PCR (qPCR).

**Figure 3:**
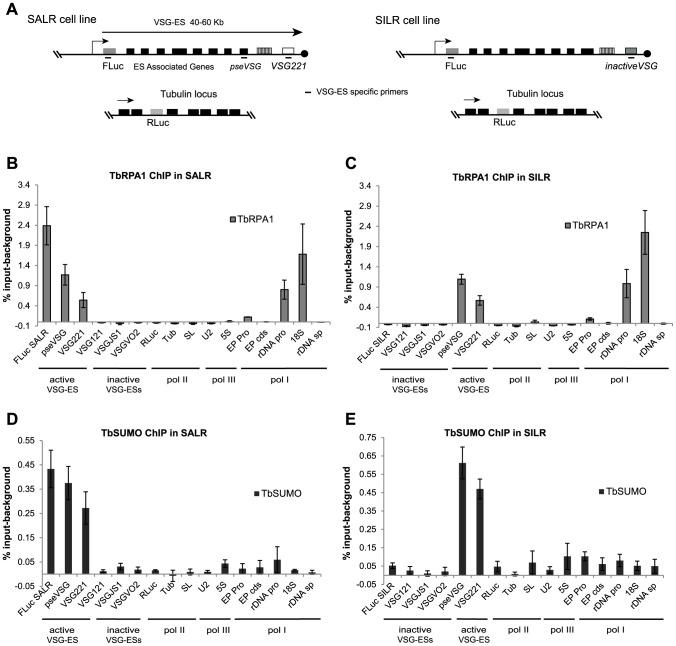
SUMOylation of chromatin-associated proteins occurs at the active *VSG-*ES in the bloodstream form. (A) Schema of tagged cell lines used in ChIP experiments. SALR cell line: *firefly luciferase* gene (FLuc) inserted downstream of the active *VSG221*-ES promoter and SILR: FLuc gene inserted downstream of an inactive *VSG*-ES. *Renilla*-luciferase reporter gene (RLuc) was inserted in the tubulin locus of both cell lines, as RNA pol II control. Fragments amplified by qPCR using ChIPed DNA are indicated (primers are listed in the Table S1). (B), (C) TbRPA1 ChIP analysis in SALR and SILR shows the occupancy of RNA pol I in these cell lines. High TbRPA1 enrichment is found in SALR along the active *VSG*-ES at FLuc gene, pseudo-VSG (pseVSG) and the active *VSG221* in contrast to inactive *VSGs*. Similar occupancy is detected in SILR except for the FLuc gene inserted in an inactive *VSG-ES* promoter of the BES5 in this cell line. The RNA pol I occupancies between FLuc SALR and FLuc SILR were found significantly different (p-value<0.01). Sequences present in rDNA promoter and 18S gene transcribed by RNA pol I were analyzed as positive controls and RNA pol II or pol III transcribed genes as negative controls. (D), (E) ChIP analysis using anti-TbSUMO mAb in SALR and SILR cell lines. Enrichment of SUMOylated proteins is found at the active *VSG*-ES chromatin from the promoter to the telomeric *VSG221*. SUMO ChIP levels between active (FLuc SALR) and inactive (FLuc SILR) reporters are significantly different (p-value<0.01). Similarly, SUMO enrichment on the active *VSG221* versus the inactive *VSGs* (*121*, *JS1* and *VO2*) is significantly different (p-values<0.05). ChIP analyses show the average from at least three independent experiments with standard error (SE). Data are represented as percentage of input immunoprecipitated (% input) after background subtraction of the negative control ChIP using the pre-bleed antiserum. Tubulin (Tub), Splicer Leader (SL), EP Procyclin promoter (EP pro), Procyclin gene (EP cds), rDNA promoter (rDNA pro), rDNA spacer (rDNA sp).

To overcome the problem of highly homologous sequences among different *VSG*-ES promoter regions [3], ChIP experiments were performed using two cell lines containing the *Firefly*-luciferase (*FLuc*) reporter gene inserted 400 bp downstream of the *VSG*-ES promoter in an active (SALR) [14], or inactive (SILR) transcriptional state. The SILR cell line contains the same *Fluc* cassette than the SARL cell line but downstream of a silent *VSG*-ES promoter (BES5, VSG800), as revealed by reporter activities and sequence analysis (see Supporting Information Text S1). In addition, to monitor a RNA pol II transcribed locus, the *Renilla*-luciferase (*RLuc*) reporter gene was inserted within the tubulin locus in both cell lines (Figure 3A). ChIP analysis using anti-TbRPA1 showed that the *VSG*-ES chromatin is highly enriched in RNA pol I in the active transcriptional state, in contrast to the inactive *VSG*-ES with immunoprecipitation levels close to the background (Figures 3B and 3C). The high enrichment of TbRPA1 at the active *VSG*-ES compared to inactive was demonstrated using the unique sequences (*FLuc*) inserted downstream of the promoter. TbRPA1 immunoprecipitated 42-fold higher at the *FLuc* the active *VSG*-ES (2.52% input) compared to *FLuc* in the inactive *VSG*-ES (0.06% input). TbRPA1 levels at additional unique sequences such as the pseudo-*VSG* and the telomeric *VSG*221 showed that the active *VSG*-ES chromatin is highly occupied by the TbRPA1 (Figures 3B and 3C). The differences of the TbRPA1 occupancy between the active and inactive *VSG*-ES sequences support a transcription initiation control as one of the mechanisms involved in *VSG*-ES monoallelic expression.

Next, we investigated the presence of chromatin-associated SUMOylated proteins within the *VSG*-ES locus by ChIP using the anti-TbSUMO mAb. Interestingly, we detected SUMOylated proteins enriched at the entire active *VSG*-ES transcription unit, from sequences downstream of the promoter to the telomeric *VSG* gene (Figures 3D and 3E). SUMOylated proteins were immunoprecipitated more efficiently at the reporter inserted downstream of the active *VSG*-ES promoter (*FLuc* SALR: 0.43% input-background) than at the inactive (*FLuc* SILR: 0.05% input-background) and the difference was statistically significant (p value<0.01). Similarly, the active *VSG*221 was significantly immunoprecitated while other telomeric *VSG* genes such as *VSG121*, *VSGVO2* and *VSGJS1*, which include also basic copies, were very low, near to background levels (Figures 3D and 3E). Furthermore, significant SUMOylation level was not detected at other RNA pol I-transcribed loci (rDNA or EP *procyclin*), nor at other RNA pol II or RNA pol III loci analyzed (Figures 3D and 3E).

**Figure 4:**
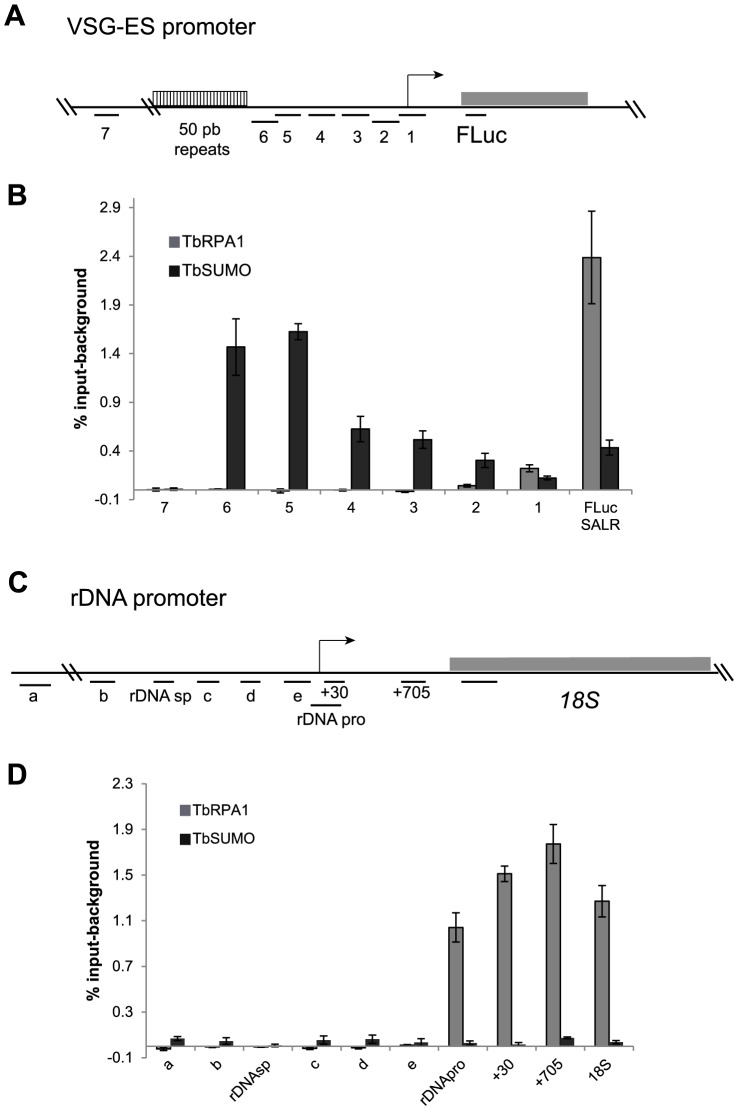
Chromatin upstream of active *VSG*-ES promoter is highly enriched for SUMOylated proteins. (A) Schema of *VSG*-ES promoter region mapping indicating fragments amplified by qPCR (See Table S1 and Figure 3A). (B) ChIP analysis of sequences upstream of the *VSG*-ES promoters using anti-TbRPA1 and anti-TbSUMO antibodies. Fragments 5 and 6 are highly enriched for SUMOylated proteins and the fragments 1–4 show moderate enrichment. In contrast, TbRPA1 is no detected in the region upstream and enriched at the promoter and downstream sequences. PCR fragment 4 from genomic and ChIPed DNA was cloned and sequenced showing that SUMO-ChIPed chromatin corresponds to the active *VSG221*-ES (See Figure S5B). (C) Schema of ribosomal DNA (rDNA) promoter showing mapped fragments amplified by qPCR (primers listed in Table S1). (D) Chromatin upstream of the rDNA promoter is not significantly SUMOylated. ChIP analysis of rDNA promoter region shows no SUMO enrichment detected above background. The results show the average from at least three independent experiments with standard error (SE). Data are represented as percentage of input immunoprecipitaded (% input) after background subtraction of the pre-bleed antiserum ChIP.

In other eukaryotes, SUMOylated proteins were detected at RNA pol II promoters and play important roles in their activity [36], [37]. Thus, we investigated the presence of SUMOylated proteins in the chromatin upstream of the *VSG*-ES promoters (Figure 4A). ChIP-qPCR analysis revealed a high enrichment of SUMOylated chromatin-associated proteins upstream of the promoter region, which was notably higher in the fragments 6 and 5 (1.5% and 1.6% input-background) (Figure 4B). As a negative control, we compared with fragment 7 upstream of the 50 bp repeats, which showed no significant enrichment (0,01% input) (Figure 4B). The trypanosome genome contains at least 15 different *VSG*-ESs with highly conserved sequences at the promoter region, suggesting that the primers used for ChIP qPCR may anneal on many different *VSG*-ESs. Relative quantification revealed that sequences 4, 3 and 1 were highly conserved in many *VSG-*ESs (Figure S5A), suggesting that SUMO ChIP values upstream of the promoter (fragments 2, 3 and 4 in Figure 4B), represented as percentage of input, were in fact underestimated. To confirm this hypothesis, we cloned and sequenced PCR fragments from the region 4 using ChIPed and genomic DNA as templates. Sequences obtained from genomic DNA yielded 14 different sequences including one from the *VSG*221-ES, indicating these PCR primers amplify most of the *VSG*-ESs in the genome. However, analysis of the anti-TbSUMO ChIPed fragments identified 11 sequences identical to the active *VSG*221-ES promoter region, and 7 sequences 99% homologous (Figure S5 B). These results together indicate that the chromatin upstream of the active *VSG*-ES promoter is highly enriched in SUMOylated proteins.

We also analyzed in detail chromatin SUMOylation along the rDNA promoter region, however no significant levels were detected in any of the positions analyzed, including the non-transcribed upstream spacer and the coding region for the 18S rRNA (Figures 4C and 4D).

### TbSIZ1 is required for the SUMOylation of chromatin-associated proteins detected in the active *VSG*-ES

**Figure 5:**
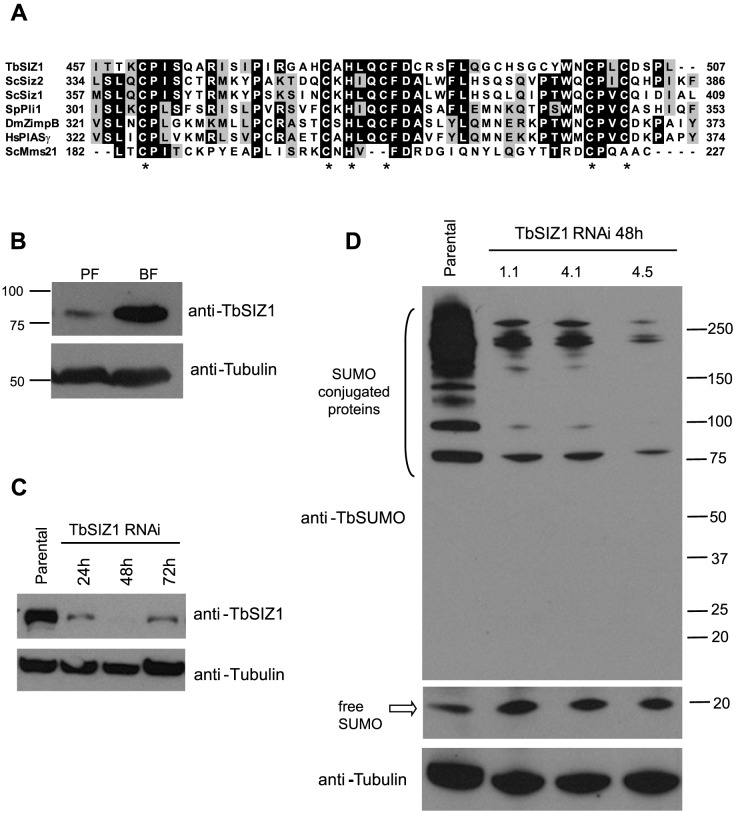
Identification and functional analysis of the SUMO ligase TbSIZ1. (A) TbSIZ1 contains a conserved SP-RING domain characteristic of SUMO E3 ligases. Sequence alignment of SP-RING domains of *T. brucei* SIZ1, *S. pombe* Plip1, *S. cerevisiae* Siz1, Siz2, *D. melanogaster* Zimp-B, *Homo sapiens* hPIASy and *S. cerevisiae* Mms21. (Accession numbers Tb927.9.11070, CAA22599.1, NP_014799, NP_010697, AAD29288, Q8N2W9, AAA20471.1 respectively). Identical residues are shaded in black and similar residues in grey. Asterisks indicate the positions of the residues forming the C2HC3 conserved SP-RING domain. Alignments were performed with BioEdit Sequence Alignment Editor and ClustalW. (B) TbSIZ1 is differentially expressed in two developmental stages of *T. brucei*, bloodstream form (BF) and procyclic form (PF). Western blot analysis using a monoclonal antibody (mAb) generated against TbSIZ1 (7G9B4). Cell extracts were prepared in the presence of 20 mM NEM. Anti-tubulin was used as loading control. (C) Depletion of TbSIZ1 in bloodstream form by RNAi at several time points after induction. Western blot analysis using anti-TbSIZ1 mAb 7G9B4 and anti-tubulin as loading control. (D) TbSIZ1 promotes SUMOylation in vivo. SUMO conjugates are reduced upon TbSIZ1 knockdown. Total cell extracts from parental and three independent clones were analyzed by Western blot analysis using anti-TbSUMO mAb, 48 h after induction of the TbSIZ1 RNAi. Anti-tubulin was used as a loading control. See also Figure S7B for IF analysis of TbSIZ1 RNAi.

We previously proposed that TbRPB7 functions in trypanosome RNA pol I transcription by recruiting transcription or RNA processing factors to the *VSG*-ES chromatin [14]. Thus, we searched for TbRPB7-interacting proteins by a yeast two-hybrid screen (Hybrigenics). This approach detected several putative interacting proteins, including a topoisomerase, a ubiquitin ligase and a protein with a SP-RING conserved domain characteristic of SUMO E3 ligases [38], which we named TbSIZ1 (Tb927.9.11070), an ortholog of yeast SIZ and mammalian PIAS. Sequence alignment of SP-RING domains of previously characterized SUMO E3 ligases revealed a significant conservation with TbSIZ1 (Figure 5A). We developed a mouse monoclonal antibody (7G9D4) anti-TbSIZ1 that allowed us to identify TbSIZ1 as a 72 kDa protein highly expressed in the infective bloodstream form of the parasite (Figure 5B).

Subcellular localization analysis detected TbSIZ1 mainly in numerous nuclear foci (Figure S6A), similar to the pattern described for other SUMO E3 ligases in other eukaryotes [39], [40]. Although TbSIZ1 was not enriched in a single nuclear area as the HSF, we investigate a possible colocalization with the active VSG-ES. Statistical analysis using the cell line with the GFP-tagged active VSG-ES showed that TbSIZ1 was associated with this locus in 87% of G1 cells (1K1N cells), while in G2 and pre-mitotic cells this percentage was reduced to 40% (2K1N cells) (Figure S6B). The lack of significant colocalization of TbSIZ1 with the rDNA locus suggests the interaction of TbSIZ1 and the active VSG-ES is not accidental (Figure S6B).

SUMO E3 ligases are important for the efficient transfer of a SUMO group from the conjugating enzyme E2 to specific substrates [22]. To characterize the function of TbSIZ1 we generated bloodstream form cell lines where depletion of TbSIZ1 was performed by RNA interference (RNAi). Western blot analysis confirmed TbSIZ1 depletion after 48 h of RNAi induction (Figure 5C), while only a minor effect in cell growth or cell cycle progression was detected (Figure S7A). However, at 72 h after induction of the TbSIZ1 RNAi the protein level increased suggesting TbSIZ1 depletion is partial and transitory (Figure 5C). Importantly, TbSIZ1 partial depletion reduced the signal of SUMO-conjugated proteins by Western blot analysis (Figure 5D), indicating that TbSIZ1 is a functional SUMO E3 ligase.

Consistently with Western analysis (Figure 5D), depletion of TbSIZ1 also reduced the nuclear signal of SUMO conjugates analyzed by IF using the anti-TbSUMO mAb (Figure S7B). Unfortunately, while the nuclear signal of TbSUMO was significantly reduced by TbSIZ1 depletion, we failed to completely eliminate the HSF signal in the nucleus. TbSIZ1 depletion functioned with variable penetrance in each cell, since the SUMO conjugated protein signal was reduced with different efficiency. SUMOylation is essential in *T. brucei* since SUMO depletion by RNAi induced deregulation of the cell cycle [29]. Notwithstanding, we decided to analyze the stability of the HSF signal in the nucleus upon TbSUMO RNAi. Similar to TbSIZ1 depletion, TbSUMO RNAi clearly reduced SUMOylation in the nucleus, however the HSF was weaker but still detected (Figure S7B).

**Figure 6:**
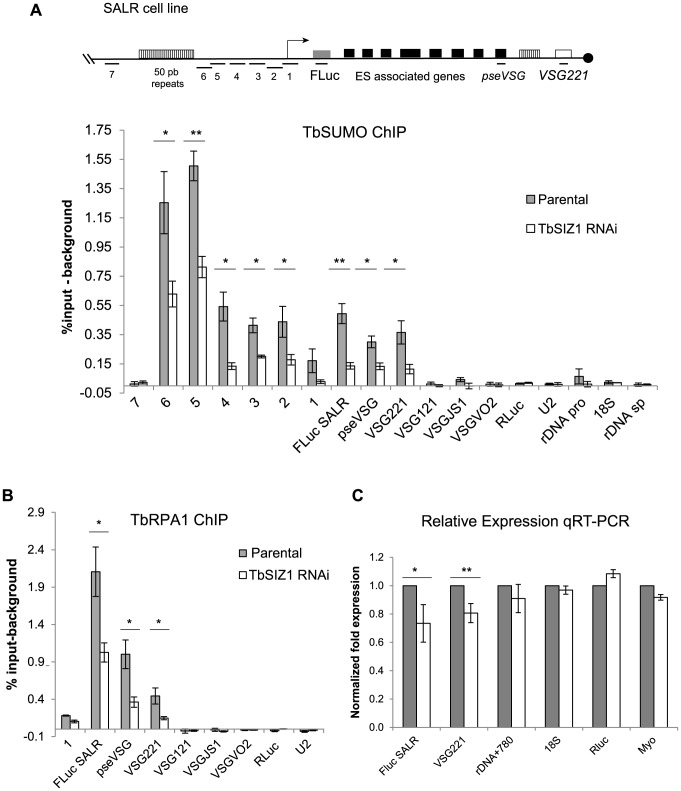
SUMOylation of chromatin-associated proteins by TbSIZ1 is important for efficient recruitment of RNA pol I and *VSG*-ES expression. (A) Reduced *VSG*-ES chromatin SUMOylation upon TbSIZ1 depletion. ChIP analysis carried out in TbSIZ1 depleted cells (48 h) and parental cell line (SALR) shows that SUMOylated chromatin is significantly reduced along all active *VSG*-ES, from the upstream promoter to the active *VSG221* gene. (B) Reduced RNA pol I occupancy upon TbSIZ1 depletion. Occupancy of RNA pol I was determined by TbRPA1 ChIP. Statistical analysis shows a significant difference of TbRPA1 levels between parental and TbSIZ1 depleted cells at the active *VSG*-ES. Data from three independent TbSIZ1 RNAi clones and parental controls are represented as percentage of input immunoprecipitated after background subtraction of the pre-bleed antiserum ChIP. The results show the average from at least three independent experiments with standard error (SE). Statistical analysis (Student's t-test), *p<0.05, **p<0.01. (C) Reduced RNA pol I occupancy results in reduced *VSG*-ES derived transcripts. Quantitative RT-PCR analysis shows reduced amounts of *FLuc* reporter gene and *VSG221* mRNA without significant effect in rDNA transcripts or RNA pol II derived transcripts *RLuc* and myosin. Results are the average from three independent clones. Data were normalized with U2 mRNA, transcribed by RNA pol III. Statistical analysis (Student's t-test) *p<0.05, **p<0.01.

TbRPB7 is required for *VSG*-ES transcription *in vivo* [14], and TbSIZ1 was identified as a TbRPB7-interacting protein, thus we decided to investigate a possible function of TbSIZ1 on *VSG*-ES chromatin SUMOylation, To do so, we performed a series of anti-TbSUMO ChIP experiments after 48 h of TbSIZ1 depletion. Upon TbSIZ1 knockdown, reduced levels of SUMO were detected at all positions along the active *VSG*-ES compared with the parental cell line (Figure 6A). The reduction of SUMOylated chromatin after TbSIZ1 partial depletion was particularly significant at the region upstream of the *VSG*-ES promoter, where the highest enrichment of SUMO conjugated proteins was detected (Figure 4B). As expected, TbSIZ1 knockdown induced no changes in chromatin SUMOylation in loci where SUMO was undetectable, such as the silent telomeric *VSG*s (Figure 6A). Altogether, these data suggest that the SUMO E3 ligase TbSIZ1 is responsible for the SUMOylation of chromatin-associated proteins detected in the active *VSG*-ES.

### RNA pol I recruitment and transcription of the active *VSG*-ES are reduced upon TbSIZ1 depletion

The detection of SUMOylated proteins associated specifically to the active *VSG*-ES chromatin, contrary to inactive *VSG*-ES, suggests a positive role of chromatin SUMOylation in transcription driven by RNA pol I in trypanosomes. To test this hypothesis, we analyzed the effect of reduced chromatin SUMOylation induced by TbSIZ1 depletion on TbRPA1 occupancy at the *VSG*-ES chromatin. ChIP values obtained using anti-TbRPA1 and chromatin isolated from TbSIZ1 depleted cells were compared with the values from the original cell line (Figure 6B). These experiments detected lower levels of TbRPA1 recruited to the active *VSG*-ES after TbSIZ1 depletion. The reduction of TbRPA1 occupancy extended from the promoter region to the telomeric *VSG*221 gene. We did not detect TbRPA1 changes in the silent telomeric *VSG*s. The RNA pol I recruited to the active *VSG*-ES after TbSIZ1 depletion was about 50% less in single copy genes (*FLuc*, Pseudo *VSG* & *VSG*221), suggesting SUMOylation of chromatin associated proteins is important to achieve full transcription of this locus.

To determine whether reduced levels of RNA pol I occupancy affect expression levels we performed RT-qPCR analysis in cells after 48 h of TbSIZ1 depletion. We detected reduced levels of the *FLuc* and *VSG*221 mRNAs, without a significant effect in RNA pol II-transcribed *RLuc* or myosin genes or in U2 transcribed by RNA pol III (Figure 6C). Importantly, we also analyzed rDNA transcription driven by RNA pol I, but no significant changes were detected in either the mature 18S or the pre-spliced rRNA780 RNAs (Figure 6C). These results suggest that TbSIZ1-mediated SUMOylation of chromatin-associated proteins positively regulates *VSG*-ES transcription.

To further investigate SUMOylation in *VSG*-ES expression and to rule out the possibility of a SUMO-ligase independent function for TbSIZ1, we decided to analyze the effect of inducing a global reduction in SUMO levels. Thus, we generated cell lines where depletion of either TbUBC9 (E2 conjugase) or TbSUMO was performed by RNAi. TbUBC9 depletion reduced the SUMO-conjugated proteins very efficiently as detected by Western blot analysis (Figure S8). Depletion of TbUBC9 protein levels was confirmed using a mouse antiserum we developed against recombinant TbUBC9 (Figure S8).

**Figure 7:**
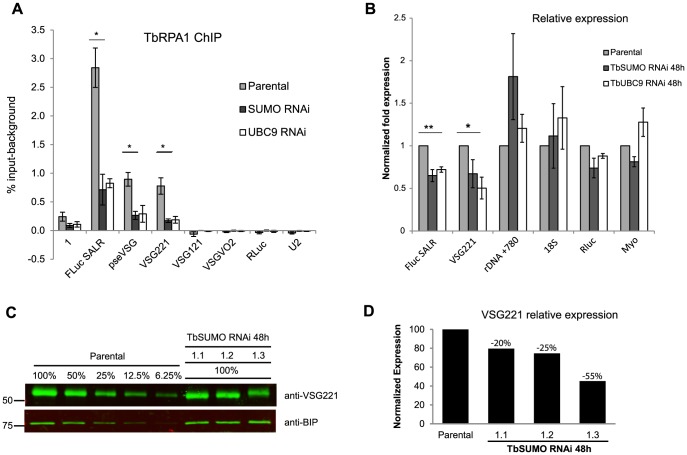
TbSUMO depletion results in reduction of VSG mRNA and protein. (A) Reduced RNA pol I occupancy upon TbSUMO and TbUBC9 depletion. TbRPA1 ChIP analysis was carried out in 48 h RNAi induced cell lines and parental cell line (SALR). Statistical analysis shows significant difference of TbRPA1 levels between parental and TbSUMO or TbUBC9 depleted cells at the active *VSG*-ES. Data from two independent clones are represented as percentage of input immunoprecipitated after background subtraction of nonspecific antiserum ChIP. Statistical analysis (Student's t test) *p<0.05,**p<0.01. (B) Reduced *VSG*-ES transcripts upon TbSUMO and TbUBC9 48 h RNAi. Quantitative RT-PCR analysis shows reduced amounts of FLuc reporter gene and VSG221 mRNA. Results from two independent clones. Data were normalized with U2 mRNA, transcribed by RNA pol III. Statistical analysis (Student's t test) *p<0.05,**p<0.01. (C) Quantitative Western blots analysis of VSG expression in three independent TbSUMO RNAi clones using IR fluorescence. Anti-VSG221 and anti BiP antibodies were incubated with two pieces from the same blot and developed using goat anti-rabbit IgG 800 Dylight (Thermo-Fisher). A standard curve based on BiPnormalized anti-VSG221 signal intensity was generated using different concentrations of parental cell extracts (R2 = 0.99). Standard curve regression was used to determine the VSG221 expression levels in TbSUMO-depleted cell lines. (D) Histogram showing the quantification of VSG221 expression relative to the parental cell line. Membrane was scanned using an LI-COR Odyssey and analyzed using Odyssey IR imaging software 3.0.42.

Cells depleted of SUMO-conjugated proteins by either TbSUMO or TbUBC9 knockdown analyzed by ChIP using anti-TbRPA1 showed a significant reduction in RNA pol I occupancy in the *VSG*-ES (Figure 7A). TbRPA1 recruitment was decreased along the entire *VSG*-ES locus without increasing the occupancy in the silent telomeric *VSG*s. The reduction of the RNA pol I occupancy upon TbSUMO or TbUBC9 depletion correlated well with a decrease of mRNA derived from the active *VSG*-ES, while ribosomal RNA levels were not reduced (Figure 7B). TbSUMO or TbUBC9 are essential genes, however their depletion has a greater effect on the *VSG*-ES mRNA levels as compared to RNA pol II-derived mRNAs (Figure 7B). These results altogether suggest that SUMOylation of chromatin-associated proteins is important for active *VSG*-ES expression.

Next, we performed quantitative Western blot analysis of VSG expression after SUMO depletion to investigate whether VSG protein levels were affected. The VSG221 expression level was analyzed using anti-BiP antibody as loading control from three independent TbSUMO RNAi clones (Figures 7C and 7D). Quantification of VSG221 expression relative to the parental cell line extracts suggested that VSG protein level was significantly downregulated upon SUMO depletion. The extent of reduction of VSG expression after SUMO RNAi was variable but consistent, suggesting SUMO functions positively in VSG expression.

### RNA pol I largest subunit TbRPA1 is SUMOylated in a TbSIZ1-depending manner

**Figure 8:**
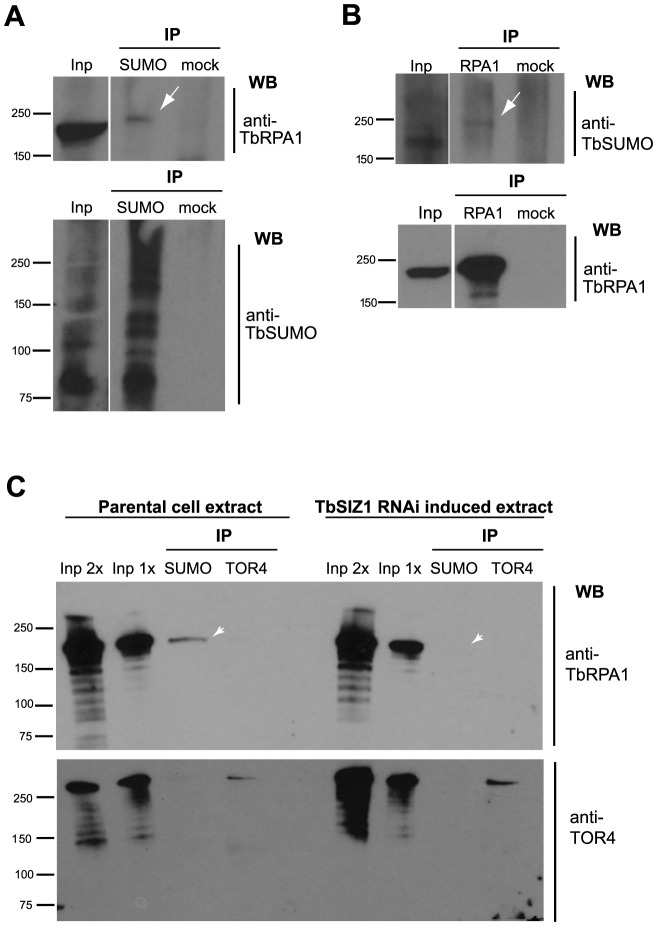
TbRPA1 is SUMOylated in *T. brucei*. (A) Immunoprecipitation (IP) of SUMOylated proteins revealed that TbRPA1 is SUMOylated. Nuclear fraction from BF cell extracts was lysed in urea containing buffer. SUMOylated proteins were immunoprecipitated with anti-TbSUMO mAb or unspecific antiserum (mock) and probed with anti-TbRPA1 (arrow). As control the same blot was probed with anti-TbSUMO (bottom) (B) Reciprocal IP experiment was performed using anti-TbRPA1 antiserum and probed with anti-TbSUMO mAb. As control the same blot was probed with anti-TbRPA1 (bottom). Loading information: input (Inp): 0.3%, IPs: 50%. (C) TbRPA1 is SUMOylated in a TbSIZ1 dependent manner. Immunoprecipitation experiments were carried out using protein extracts from the parental cell line and compared with a TbSIZ1 depleted extract (48 h RNAi induced). SUMOylated proteins were immunoprecipitated with anti-TbSUMO mAb or anti-TOR4, as control. Western blot with anti-TbRPA1 shows a reduction of the SUMOylated TbRPA1 detected upon TbSIZ1 depletion (arrowhead). Loading information: input 2× (Inp 2×): 0.1%, input 1× (Inp 1×): 0.05%, IPs: 50%.

The detection of the HSF in the nucleus (Figure 2) and the high occupancy of SUMOylated proteins at the *VSG*-ES chromatin (Figures 3 and 4) suggest that a large number of SUMOylated proteins occur at this site, similar to Protein Group SUMOylation described previously [41]. Identification of the SUMO-conjugated proteins in the HSF is beyond the scope of this work, however the largest subunit of the RNA polymerase I is an obvious candidate since it is SUMOylated in other eukaryotes [42]. To investigate a possible TbRPA1-SUMO conjugation, we performed IP assays utilizing anti-TbSUMO mAb and affinity-purified TbRPA1 antiserum under denaturing conditions, which preserve SUMO conjugation (see Supplementary Information Text S1). IP experiments revealed that TbRPA1 is SUMOylated as shown by Western analysis using anti-TbRPA1 on a SUMO IPed extract (Figure 8A). The reciprocal experiment using anti-TbSUMO antibody on anti-TbRPA1 IPed extract reproducibly detected TbRPA1-TbSUMO conjugates (Figure 8B). The low detection of SUMO-conjugated TbRPA1 is probably due to the large number of SUMOylated proteins and the small percentage of TbRPA1 that is SUMOylated, as occurs with other SUMO targets in eukaryotes [33]. This result suggests that under normal growth conditions a fraction of TbRPA1 is SUMOylated. While this may contribute to the SUMOylation we detected by ChIP on the *VSG*-ES chromatin, SUMOylation is also detected upstream of the promoter (Figure 4B), suggesting additional SUMOylated chromatin proteins occur at this region.

We addressed whether the fraction of SUMO-conjugated TbRPA1 is the one that resides in the extra-nucleolar body ESB. To do so, we used the Proximity Ligation Assay (PLA) (O-link Bioscience). This technique exploits the distance requirements of a PCR reaction by linking two primers to the two secondary antibodies. The PLA assays showed that the fraction of TbRPA1 that is SUMOylated resides at an extra-nucleolar site (Figure S9). This result, together with the IF colocalization analysis of the HSF with both the active *VSG*-ES locus and the TbRPA1 (Figures 2A and 2B) suggest that SUMOylated TbRPA1 occurs at the nuclear body ESB.

Finally, we wished to investigate whether SUMOylation of TbRPA1 is mediated by TbSIZ1. To characterize a possible function of TbSIZ1 in TbRPA1 SUMOylation we performed a series of co-IP experiments using protein extracts isolated from TbSIZ1 depleted cells and compared to the parental cell line. Figure 8C shows that TbSIZ1 depletion significantly reduced the amount of TbRPA1 IPed using anti-TbSUMO antibody. This result suggests that TbSIZ1 mediates SUMO targeting of TbRPA1.

## Discussion

The importance of nuclear bodies and the three-dimensional organization of chromosomes in the regulation of gene expression is becoming evident in eukaryotes [43]. In trypanosomes, it was suggested that the recruitment of a single Variant Surface Glycoprotein Expression Site (*VSG*-ES) telomeric locus to a discrete, RNA pol I-containing nuclear body (ESB) underlies the mechanism responsible for *VSG* monoallelic expression [4], [5], [44]. Here, by nuclear localization analysis using 3D microscopy, we describe a highly SUMOylated focus (HSF) (Figure 1C). The nuclear position of the HSF partially colocalizes with the active *VSG*-ES locus and the nuclear body ESB (Figure 2). Unfortunately, our attempts to completely eliminate the HSF in the nucleus by TbSUMO or TbSIZ1 RNAi were unsuccessful (Figure S7B). Previous evidence whereby SUMO modifies the interaction properties of conjugated proteins and affects their subnuclear localization [45] suggests that SUMOylation of nuclear proteins at the HSF might be involved in the nuclear body ESB regulation in trypanosomes.

SUMO-conjugated proteins are localized to the active *VSG*-ES chromatin, in contrast to any other loci examined (Figure 3). We have investigated the possibility that SUMOylated proteins associate with other loci transcribed by RNA pol I. SUMOylated chromatin was not detected at the rDNA or EP procyclin loci. These results suggest that the association of SUMO-conjugated proteins to chromatin is a distinct feature of the *VSG*-ES regulation.

SUMOylation of chromatin-associated proteins at the active *VSG*-ES extends from ∼1 Kb upstream of the promoter down to the telomeric *VSG*, while SUMO was not detected at silent *VSG*-ESs or *VSG* basic copies chromatin (Figures 3 and 4). Whilst SUMOylation has been classically associated with transcriptional repression [46]–[48], there is some evidence that SUMOylation can also function as a transcriptional activator, particularly to modify gene-specific transcription factors or co-regulators [28], [49]. In HeLa cells, SUMO-1 was found at the chromatin just upstream of the transcription start site on many of the most active genes [36]. Depletion of SUMO-1 resulted in down regulation of transcription supporting the idea that marking of promoters by SUMO-1 is associated with transcriptional activation [36]. PIAS E3 ligases function as enhancers of c-Myb activity in active nuclear RNA pol II foci [39]. In trypanosomes, the transcriptionally active *VSG*-ES promoter and the nuclear body ESB are identified here as being highly SUMOylated (Figures 1C and 2).

TbSIZ1 is the first SUMO E3 ligase functionally analyzed in *T. brucei*. It contains a conserved SP-RING domain essential for the ligase activity described previously in other eukaryotes [38]. TbSIZ1 depletion has a mild effect on cell growth and cell cycle progression (Figure S7A). This result is similar to other SUMO ligases, such as *S. pombe* Pli1 and *S. cerevisiae* Siz1 and Siz2, for which deletion does not affect cell growth [20], [40]. Interestingly, TbSIZ1 depletion reduced some SUMO-conjugated protein bands more efficiently than others analyzed by Western blot (Figure 5D). This supports the idea of specificity of TbSIZ1 substrates, similarly to the role of previously described SIZ/PIAS E3 ligases [21].

In the present work, we show that TbSIZ1 functions *in vivo* as a SUMO E3 ligase of chromatin-associated proteins detected at the active *VSG*-ES chromatin by ChIP. Depletion of TbSIZ1 causes reduction in SUMOylation of the active *VSG*-ES with a concomitant reduction in RNA pol I occupancy and transcriptional activity (Figure 6B and C). We ruled out the possibility of a SUMO ligase independent function of TbSIZ1 by TbSUMO or TbUCB9 RNAi experiments, which also reduced both RNA pol I recruitment and *VSG*-ES expression (Figure 7). This finding is similar to observations in yeast, where SUMOylation of chromatin-associated proteins in actively transcribed genes is dependent on the E2 conjugating enzyme Ubc9 [37]. Interestingly, we did not detect significant levels of SUMOylated chromatin in the other RNA pol I-driven control loci as rDNA, EP or silent VSG-ESs loci, suggesting that SUMO plays a distinct function in *VSG*-ES positive regulation.

We showed that the constitutive rDNA promoters have no detectable levels of SUMOylated chromatin (Figure 4D), contrary to the switchable *VSG*-ES promoter, which is highly SUMOylated only in the active transcriptional state. Activation of inducible promoters has been shown to result in chromatin SUMOylation, suggesting that gene activation involves SUMOylation of promoter-bound factors [37]. Our results suggest a function of SUMO in *VSG*-ES active transcription, since upon SUMO depletion by TbSIZ1, TbUBC9 or TbSUMO RNAi, both recruitment of the RNA pol I at the *VSG*-ES promoter and *VSG*-ES derived transcripts are reduced.

The finding that SUMOylation is important for *VSG*-ES expression suggests that factors previously implicated in *VSG* regulation maybe modified by SUMOylation. An obvious candidate as SUMO substrate is the RNA pol I complex, responsible for *VSG* transcription. In other eukaryotes, several subunits of the RNA pol I, including RPA1, were described to be SUMOylated in large scale proteomics analyses [42], [50]. Indeed, we find by IP experiments that TbRPA1 is SUMOylated (Figure 8). However, the high SUMO enrichment detected 1 Kb upstream of the *VSG-*ES promoter cannot be accounted for TbRPA1. Thus, SUMOylated proteins detected upstream of the *VSG*-ES promoter may include transcription factors or structural components of chromatin, similar to what has been described in other eukaryotes [47].

Simultaneous SUMOylation of Protein Groups by modification of multiple targets providing synergy in a specific process has been recently described for DNA repair [41], [51]. Proteomic studies have shown that several proteins in the same complexes or biochemical pathways are SUMOylated [42], [50]. Protein group SUMOylation may also be associated with a specific subnuclear localization of SUMOylated proteins [52]. The HSF is frequently larger than the ESB detected using anti-TbRPA1, suggesting that additional factors involved in processes previously associated with VSG expression, such as transcription elongation and mRNA maturation, may be present in the HSF [2]. Our data showed that RPA1 immunoprecipitated 42-fold higher at the active VSG-ES as compared to a single inactive VSG-ES, however we still detect some TbRPA1 at the inactive VSG-ES site (FLuc SILR: 0.061% input, before removing background). Possibly, this polymerase in the inactive *VSG*-ESs promoter region is enough to produce detectable mRNA described recently [53]. It seems likely that the HSF described in this work represents a group of post-transcriptionally modified proteins, as Protein Group SUMOylation, functionally associated with *VSG*-ES transcription initiation, elongation and mRNA maturation.

Some of the proteins involved in the regulation of antigenic variation and *VSG*-ES expression in trypanosomes have been previously described as SUMO targets in other eukaryotes. Among the possible chromatin-associated factors that could be SUMOylated at the active *VSG*-ES is the architectural chromatin protein TDP1, which was reported to be enriched at the active *VSG*-ES and rDNA, facilitating RNA pol I transcription [13]. The yeast ortholog Hmo1 has been recently identified by proteomic analysis as SUMO-conjugated protein [50]. Thus, it seems possible that the fraction of TDP1 at the ESB is SUMOylated in the HSF.

Recent data showed that Cohesin subunits Smc1/3 and Scc1/3 are SUMOylated in yeast [54]. In *Trypanosoma cruzi* SMC3 was also identified as a SUMOylated protein by proteomic approaches [31]. We have recently identified Cohesin complex as a factor involved in *VSG*-ES switching [34]. Preliminary results suggest that SMC3 is SUMOylated in *T. brucei*, however and contrary to TbRPA1, SUMO is not targeted to TbSMC3 by TbSIZ1 (manuscript in preparation).

ChIP experiments showed highly-enriched SUMOylated chromatin upstream of the *VSG*-ES promoter (Figure 4), suggesting structural components of chromatin might be also targets for SUMO at this particular location. In trypanosomes, post-translational histone modifications are being associated with repression of silent *VSG*-ESs (see for review [55]). Histone SUMOylation is associated with transcriptional repression in *S. cerevisiae*, where all four core histones are SUMOylated [47]. However we describe the lack of SUMOylated chromatin at silent *VSG*-ESs, while the active *VSG*-ES chromatin is highly enriched in SUMO. Our results show SUMO as a post-translational modification of proteins associated with the active transcriptional state of the *VSG*-ES.

Ever since the finding that the ESB is associated with *VSG*-ES monoallelic expression [4], we and others have searched for a particular factor located exclusively at this unique nuclear body. However, specific post-translational modifications of common factors may also account for this body. Here we report that TbRPA1, the largest subunit of RNA pol I, is SUMOylated by TbSIZ1 (Figure 8C), in addition IF colocalization analysis and PLA (Figures 2B and S8), strongly suggest that the fraction of SUMOylated TbRPA1 resides at the ESB rather than in the nucleolus. SUMO modification has been involved in the re-localization of transcriptional regulators to different subnuclear compartments [56] and stabilizes interactions between the functionally related proteins [41]. Taken together, our results suggest a model whereby SUMOylation of chromatin-associated proteins mediated by TbSIZ1 at the active *VSG*-ES locus may function to nucleate factors to the ESB.

The complex regulation of antigenic variation involves monoallelic transcription of a single *VSG*-ES out of a multiallelic gene family at any given time. Our results show a positive mechanism via SUMOylation that marks the active *VSG*-ES chromatin. In other eukaryotes, SUMOylation of transcription factors and chromatin proteins is a negative mark that represses gene expression in most cases. The surprising observation about the specificity of chromatin SUMOylation for the active transcription state in an early-branched eukaryote suggests that the post-translational modification of proteins by SUMO play a basic role in the positive regulation of transcription in eukaryotes. Chromatin SUMOylation as an epigenetic mark for the monoallelically expressed *VSG*-ES could apply more widely to the regulation of antigenic variation in other protozoan parasites [57], [58].

## Materials and Methods

### Trypanosomes strains and cell lines

*T. brucei* bloodstream form (Lister 427, antigenic type MiTat 1.2, clone 221a) and 427 procyclic form were used in this study. The dual-reporter SALR cell line in the bloodstream single-marker cell line was previously described [14], [59]. SALR dual reporter cell line contains a Firefly Luciferase (*FLuc*)-reporter integrated 405 bp downstream of the active ES promoter, and *Renilla*-Luciferase reporter integrated in the tubulin locus. The generation of dual-reporter SILR cell line was similar to the SALR cell line, but the same construct containing the *Fluc* gene was integrated downstream of an inactive ES promoter (see Supporting Information Text S1). The insertion site was identified by PCR and sequencing of the flanking luciferase region from SALR and SILR genomic DNA confirming *FLuc* is inserted in the active *VSG221*-ES in SALR, and in SILR downstream of the inactive *VSG*-ES promoter BES5/TAR98 VSG800/427-18 [3]. The *VSG221*-ES and rDNA GFP-LacI tagged cell lines have been previously described [4], [35]. The cell line expressing a YFP-TbRPB5z fusion was described before [35].

### Recombinant proteins and monoclonal antibodies

N-terminal fragment of TbSIZ1 (Tb927.9.11070), full-length of TbSUMO (Tb927.5.3210) and TbUBC9 (Tb927.2.2460) were amplified by PCR (See primers in Table S1). PCR products were cloned into *BamH*I and *Hind*III sites of pET28a vector (Novagen), and expressed as an N-terminal His tag. Purification of recombinant proteins was performed using NI Sepharose Fast Flow 6 (GE Healthcare). Purified recombinant proteins were inoculated in mice and used to generate anti-TbSIZ1 (7G9B4) and anti-TbSUMO (1C9H8) monoclonal antibodies (mAb), using standard procedures. Hybridomas were first screened against the recombinant proteins by ELISA and later confirmed by western blot analysis using trypanosome protein extracts since recognized a single protein of the expected size. Hybridomas 7G9B4 and 1C9H8 cell lines were grown as ascites. Anti-TbUBC9 mouse antiserum was generated using purified recombinant his-tagged TbUBC9 as antigen using standard procedures.

### Immunofluorescence

Mouse anti-TbSUMO (1C9H8) mAb (1∶2000), rabbit anti-TbRPA1 affinity-purified antiserum (1∶600) [4] and rabbit anti-GFP polyclonal (1∶5000; Invitrogen) were used as primary antibodies. Goat anti-mouse and anti-rabbit Alexa 488 or 594 conjugated antibodies (Invitrogen) were used as secondary antibodies. Detailed subcellular localization and colocalization analysis was performed by deconvolution 3D microscopy as described previously [35] (see Supporting Information Text S1).

### Chromatin Immunoprecipitation (ChIP)

ChIP was performed as described previously [60] with some modifications. In brief, *T. brucei* bloodstream cultures were fixed in 1% formaldehyde at 37° for 15 min. Pellets were resuspended in 1 ml of lysis buffer per 10^8^ cells and sonicated to shear the chromatin to ∼300pb in length. Sheared chromatin was diluted 1∶5 in ChIP dilution buffer and pre-cleared with Sepharose 4B beads (Sigma). An aliquot of the input DNA (10%) was saved. 2.5 ml of pre-cleared chromatin (5×10^7^ cells per ChIP) was incubated overnight at 4°C with each antibody (6 µg of anti-TbRPA1, 60 µg of anti-TbSUMO 1C9H8, 60 µg of unspecific antiserum). Next, protein G Sepharose (Sigma) was added and incubated for 1 hr at 4°C; Immunoprecipitates were washed and eluted from the beads. Crosslinks were reversed at 65°C for 15 h. After RNase and Proteinase K treatment, DNA was extracted with phenol∶chloroform and ethanol precipitated. DNA was resuspended in 50 µl of miliQ water and analyzed by quantitative PCR (qPCR). To compare the amount of DNA immunoprecipitated to the total input DNA, 10% of the pre-cleared chromatin saved as input was processed with the eluted immunoprecipitates beginning at the crosslink reversal step. Quantitative PCR (qPCR) was performed using the SYBR green supermix (Quanta Biosciences) in a CFX96 cycler (BioRad), as described below for RT-qPCR. qPCR mixtures contained 2 µl of a 1∶5 dilution of the ChIPed DNA or a 1∶50, 1∶100, 1∶200 dilution of the input sample and 500 nM of each primer in a final reaction volume of 10 µl. All reactions were performed in duplicate and each product was verified by melting curve analysis. The PCR primers used to analyze target fragments were designed by using the Primer3 software and synthetized by Sigma, targets and sequences are listed in Table S1. Standard curves with serial dilutions of input DNA were made to determine PCR efficiency and to determine IP percentages. The relative amount of each specific PCR fragment in the ChIPed DNA and in the input DNA was calculated against the standard curve equation, next the percentage of input immunoprecipitaded was calculated. Finally the background values from unspecific antiserum (pre-bleed rabbit antiserum ChIP) were subtracted from the values obtained with the specific antibodies. Fold values were determined using the percentage of input immunoprecipitated before the background correction, since the background values were very similar between the loci to compare. Independent ChIP experiments were performed at least three times and statistical analysis (Student's t-test) was applied to compare data sets. See supplementary information (Text S1) for more details and primer sequences are provided in the Table S1.

### RNAi experiments

RNAi constructs were made using the p2T7Bla vector [14], which allows Tet-inducible expression of dsRNA from opposite T7 promoters [61]. Since most of the RNAi constructs using this vector were leaky, comparative analyses always included in addition of the dox induced (+) and uninduced (−) RNAi, the parental cell line (SALR) [14]. Fragments corresponding to 642-pb of TbSIZ1 gene, full length of TbSUMO ORF gene (345-pb) and 5′UTR TbSUMO fragment (121 bp) and full length TbUBC9 gene (594-pb) were amplified by PCR using primers described in Table S1 and cloned into *BamH*I and *Hind*III sites of p2T7Bla. The constructs were linearized and stably transfected into the dual-reporter cell line SALR [14]. dsRNA synthesis was induced by the addition of µg ml^−1^ of doxycycline. At least three independent clones from each construction were analyzed and depletion of the proteins was confirmed by Western blot using specific antibodies.

### RT-qPCR

Total RNA samples were extracted from 40 ml parasite cultures (4×10^7^ cells) using the High Pure RNA isolation Kit (Roche) and treated with integrated DNA digestion and DNase removal following manufacturer's instructions. RNA quality was verified by gel analysis, nanodrop quantification and A260/A280 ratio. cDNA was synthesized from 2 µg of RNA with the SuperScript IIII Reverse Transcriptase (Invitrogene) and random primers (Invitrogene) following manufacturer's instructions. RNA samples not treated with reverse transcriptase were used as a negative RT control and analyzed by quantitative PCR for DNA contamination assessment. Quantitative PCR was performed using the SYBR green supermix (Quanta Biosciences) in a CFX96 cycler (BioRad), using 96-well clear low profile plates, sealed with clear optical adhesive covers. PCR mixtures contained 5 µl of 2× SYBR green supermix, 500 nM of each primer and 1 µl of cDNA for single copy genes or 1 µl of a 1∶100 dilution for multicopy genes in a final reaction volume of 10 µl. All reactions were performed in duplicate and each product was verified by melting curve analysis. The PCR protocol used was 95°C for 3 min followed by 32 cycles of 95°C for 30 sec, 60°C for 30 sec, 72°C for 30 sec, then 72°C for 1 min and final melting curve from 55 to 90°C, increment 0.5°C/5 sec. Fluorescence readings were taken during the extension step. PCR primers were designed by using the Primer3 software and synthetized by Sigma. Primer targets and sequences are listed in Table S1. Standard curves for each primer pair were generated with serial dilutions of cDNA to determine PCR efficiency. The relative levels of gene expression between a given sample and the control sample (Parental cell line) were calculated using the ΔΔCT method with the Bio-Rad CFX Manager software. The U2 gene transcribed by RNA pol III was used as reference gene to normalize RNA starting quantity since it was stably expressed, invariant expression was confirmed using Myosin B or *Renilla*-luciferase (RLuc) as reference genes. Three RNAi independent clones were analyzed and statistical analysis (Student's *t*-Test) using SigmaPlot software was performed.

See the Supplemental Material and Methods (Text S1) in Supporting Information for additional protocols.

## Supporting Information

Figure S1 **Analysis of SUMOylation pattern in** ***T. brucei*** **cell extracts.** (A) TbSUMO RNAi using the 5′UTR as a different targeting sequence. Western blot analysis using the TbSUMO mAb 1C9H8 and cell extracts prepared in the presence of 20 mM NEM. (B) Effect of the de-sumoylation inhibitor N-ethylmaleimide (NEM) in BF cell extracts. Lysates were prepared with 1× protease inhibitor cocktail (Roche) and different concentrations NEM. Free SUMO is less abundant in presence of the de-sumoylation inhibitor NEM. Conversely, samples without NEM showed an increased detection of free SUMO, due to the activity of internal de-sumoylases from trypanosome protein extracts, with a concomitant reduction of SUMO conjugated proteins. (C) Western blot analysis comparing the SUMOylation pattern obtained using either anti-TbSUMO mAb 1C9H8 or anti-T.cruzi SUMO rabbit antiserum [30] [31]. Protein extracts prepared in the presence of 5 mM NEM were obtained from the parental cell line, uninduced (−) and TbSUMO RNAi cell line after 24 and 48 hours of induction. The same membrane was first incubated with anti-TbSUMO mAb 1C9H8, next de-hybridized and probed with anti-TcSUMO rabbit antiserum. The SUMO-conjugated proteins reduction detected upon TbSUMO depletion using the mAb anti-TbSUMO suggested 1C9H8 is specific antibody. Anti-tubulin was used as a loading control.
(EPS)

Figure S2 **Statistical analysis of the HSF detection in the nucleus.** (A) Immunofluorescence (IF) analysis using the anti-TbSUMO mAb showing the highly SUMOylated focus (HSF) in bloodstream form trypanosomes. (B) Statistical analysis of the HSF detection. 74.9% of the nuclei showed a single HSF (n = 349). (C) The HSF partially colocalizes with the YFP-tagged TbRPB5z in the ESB (arrow). A cell line that expresses the RNA pol I-specific subunit, RPB5z tagged with the YFP (green) was analysed by double IF with anti-TbSUMO antibody (red). Scale bars, 1 µm. (D) Statistical analysis of GFP-tagged active *VSG*-ES (aES) and GFP-tagged rDNA colocalization with the SUMO focus showed in Figure 2B and 2C. Significant colocalization of SUMOylated proteins with the active *VSG*-ES but not with rDNA locus is observed along cell cycle phases G1 (1K1N), G2 (2K1N). Data quantification from the 3D colocalization mask analysis, n = 46 cells active *VSG-*ES (aES), n = 45 (rDNA).
(EPS)

Figure S3 **HSF remains associated with the ESB through the cell cycle.** Bloodstream form cells were analyzed by indirect 3D double IF using polyclonal anti-TbRPA1 (red) and monoclonal anti-TbSUMO (green) antibodies. DNA was stained with DAPI (blue). Maximum intensity projections of three-channel 3D representative stacks of 1K1N (G1), 1K1N with enlarged kinetoplast (G1/S), 2K1N premitotic (G2) and 2K2N (post-mitotic) cells are shown. The ESB is indicated with arrowheads and the HSF with arrow. Scale bars, 1 µm.
(EPS)

Figure S4 **HSF remains associated with the active** ***VSG*** **Expression Site (ES) through the cell cycle.** GFP-tagged active *VSG*-ES promoter cells were subjected to indirect 3D IF using anti-TbSUMO mAb (red) and anti-GFP (green) rabbit antiserum (Invitrogene). DNA was stained with DAPI. Maximum intensity projections of three-channel 3D representative stacks of 1K1N (G1), 2K1N (G2) and 2K1N mitotic (M) cells are shown. The HSF is indicated with arrow and the GFP-tagged active *VSG*-ES with arrowheads. A colocalization mask (white) was calculated using ImagenJ for each non-equalized 8- byte slice and merged with both anti-TbSUMO (red) and anti-GFP (green). Complete DAPI staining (K and N) is displayed in the lower panel. Scale bars, 1 µm.
(EPS)

Figure S5 **Identification of the** ***VSG*** **-ES promoter sequences SUMOylated.** (A) Relative quantification of sequences upstream of *VSG*-ES promoters reveals different copy number of each PCR fragment described in Figure 4. Analysis by qPCR using genomic DNA as template. PCR fragments 5 and 6 only amplify one or two additional *VSG*-ES promoters besides the active *VSG221*-ES, while fragments 1–4 are highly conserved in most *VSG*-ESs. As control, we quantified *VSG* genes and other control genes. Data are represented as fold increase over the single copy gene *firefly luciferase* (FLuc). (B) Sequence alignments of fragment 4 amplified from genomic and TbSUMO ChIPed DNA. PCR products were cloned and sequenced. Clones obtained from genomic DNA yielded 14 different sequences including one from the *VSG*221-ES, while using TbSUMO ChIPed DNA, 11 sequences were identical to *VSG*221-ES and 7 showed just one (6) or two (1) different nucleotides.
(EPS)

Figure S6 **Subcellular localization of TbSIZ1.** (A) TbSIZ1 is diffusely distributed in numerous foci in the nucleus. Immunofluorescence analysis carried out using the anti-TbSIZ1 monoclonal antibody (7G9B4) (green) and DNA stained with DAPI (blue). Scale bar, 5 µm. (B) TbSIZ1 partially colocalized with the active *VSG*-ES in a cell cycle-dependent manner. Double indirect 3D-IF was performed in a cell line where the active *VSG*-ES was GFP-LacI tagged. TbSIZ1 was detected with anti-TbSIZ1 mAb (red) and the GFP-tagged active *VSG-*ES with a rabbit anti-GFP antiserum (green). Maximum intensity projections of deconvolved slices containing the GFP dot signal are shown (arrow). The colocalization mask (white) was calculated as described above. Scale bars, 1 µm. (C) Representative cell showing that TbSIZ1 does not significantly colocalize with rDNA locus. Double 3D-IF analysis was performed in a cell line where the rDNA locus was GFP-tagged (arrow). Scale bar, 1 µm. (D) Colocalization statistical analysis between TbSIZ1 and either the GFP-tagged active *VSG*-ES (aES) or the GFP-tagged rDNA locus. Partial colocalization is observed between TbSIZ1 and the active ES (mainly in 1K1N, during G1 phase) but not with rDNA locus. Data quantification from the 3D colocalization mask analysis, n = 42 cells (aES), n = 56 (rDNA).
(EPS)

Figure S7 **Functional analysis of TbSIZ1 depletion.** (A) TbSIZ1 depletion does not significantly affect cell growth (Left). Growth curves of bloodstream trypanosome cell lines show mild effect upon TbSIZ1 RNAi. Cell cycle analysis by FACS shows a slight increase of G2 cells upon 48 h of TbSIZ1 RNAi (Right). Data were collected from two independent clones. (B) Depletion of TbSIZ1 reduces the nuclear signal of SUMO-conjugated proteins by IF. Parental cell line (WT), TbSIZ1 RNAi and TbSUMO RNAi induced cells were fixed and IF was carried out with anti-TbSUMO mAb (green). Arrowheads mark the HSF still detected after TbSIZ1 and TbSUMO depletion. Images are non-deconvolved single slide. Merged with DAPI are visualized in last panels. Scale bars, 5 µm.
(EPS)

Figure S8 **Depletion analysis of TbUBC9 in bloodstream-form cells.** Western blot analysis with anti-TbSUMO mAb showing the reduction of SUMO conjugated proteins at 24 and 48 hours after induction of TbUBC9 RNAi. Cell extracts were prepared in the presence of 5 mM NEM. Anti- TbUBC9 polyclonal antibody was used to analyze the depletion of the protein and anti-tubulin as loading control.
(EPS)

Figure S9 ***In situ*** **detection of SUMOylated TbRPA1 using a Proximity Ligation Assay (PLA).** (A) TbRPA1 SUMOylation was detected by in situ PLA (red dot) in bloodstream form using rabbit anti-TbRPA1 antiserum and mouse anti-TbSUMO monoclonal antibody. Cells were fixed and subjected to PLA assay as described in Supporting Information (Text S1). Representative G1 (upper panel) and G2-M (middle panel) cells are shown. Control reaction (lower panel) without primary antibodies. DNA was stained with DAPI. (B) Statistic analysis of dot-positive cells detected using both primary antibodies anti-TbSUMO and anti-TbRPA1, and negative controls using each antibody individually. 58.71% (±2.55%) of the cells showed dot signals in the PLA assay using both primary antibodies (n = 330). (C) Statistics analysis of dot detection throughout cell cycle progression. 1K1N (G1); V shape (S/G2); 1K1N (G2). An increase of two dots was observed when cell enters in mitosis and the active *VSG*-ES duplicates.
(EPS)

Table S1 **Primers used in cloning, ChIP-qPCR and RT-qPCR.**
(DOCX)

Text S1 **Supplementary materials and methods.** Detailed description of Trypanosomes cell lines, Yeast two-hybrid (Y2H), 3D Microscopy, Chromatin, Immunoprecipitation (ChIP), Relative quantification by qPCR, FACS analysis, Cell extracts and Immunoblots, Immunoprecipitation, Quantitative Western Blots and Proximity Ligation Assay (PLA).
(DOCX)
