# Supplementary material for: SUMOylation by the E3 Ligase TbSIZ1/PIAS1 Positively Regulates VSG Expression in Trypanosoma brucei
Source: PLoS Pathog. 2014 Dec 4;10(12):e1004545. doi: 10.1371/journal.ppat.1004545 (PMC4256477; doi:10.1371/journal.ppat.1004545)
Supplement: Table S1 — Primers used in cloning, ChIP-qPCR and RT-qPCR. (DOCX) [file ppat.1004545.s010.docx]

**Table SI.** Primers used in cloning, ChIP-qPCR and RT-qPCR

| Gene or Region | Primer name | Sequence (5´-3`) |
| --- | --- | --- |
| TbSIZ1 | ZnF_ RNAi_U  ZnF_ RNAi_L | CCCGGATCCATGGTGATCCGACCTGCAA GGGAAGCTTGCCGGCTCATTGTGCTTAAGCGGTGT |
| TbSUMO | SumoRNAi Bam_U  SumoRNAi hind_L | CCCGGATCCATGGACGAACCCACTCATAACTC  GGGAAGCTTTCACGCCATGCACCAAAGACACC |
| 5´UTR SUMO | 5UTRSUMO BamU  5UTRSUMO HindL | CCCGGATCCCGAGTTGGGTTCATTTCTGAG  GGGGAAGCTTCACACACACACACGCAAAAC |
| TbUBC9 | Ubc9_U  Ubc9_L | CCCCCATGGGGATCCATGTCCGGGCTATCTTTAGCT  GGGGAAGCTTTACCCGCTTCCGGTGGTGCTT |
| TbSIZ1 (N-terminal) | TbSIZ1 Up  TbSIZ1His Low | CCCGGATCCATGGTGATCCGACCTGCAA  GGGGAAGCTTCTTATCATTGTGCTTAAGCGGTGT |
| 1 | ESMP1_U  ESMP1_L | GGTGTGGCGGACGTCTCGAAC  CCTCTAAAATACGCTCAGCCCGTCC |
| 2 | ESMP2_U  ESMP2_L | ATGAAGGTCTTGCGCTGTCT  CGCCACACCTTGAGTCTGATATCC |
| 3 | ESMP3_U  ESMP3_L | TGTTGTTGCAGTATGTTTTTCTTC AGACAGCGCAAGACCTTCAT |
| 4 | ESMP4_U  ESMP4_L | CGGAGAATATTTCGGATGC  AATCGTTACGGCCAAATTCA |
| 5 | ESMP5int_U  ESMP5_L | TGTTGTGCTTTCTAACACTTCCTT ATTCCTCCCCACAGAAAGA |
| 6 | ESMP5_U  ESMP5int_L | TTTGTTTGTTTCATGTTTTTGTG AAGGAAGTGTTAGAAAGCACAACA |
| 7 | ESMP9_U  ESMP9_L | TGGTTGCAGTTATGGAGCAG  CCAACCCTCAAGCAGGATAA |
| VSG221 | VSG221_U  VSG221_L | AGCTAGACGACCAACCGAAGG  CGCTGGTGCCGCTCTCCTTTG |
| PseVSG | 221pseudo_U  221pseudo_L | CAAGCATTACCAGAGAAGT  CGTCATTCAGTTTCCTTATT |
| VSG121 | VSG121_U  VSG121short_L | CCTGACATCGGACGGTAAC  TGGTCGTATTTGCCTTCCTT |
| VSGJS1 | VSGJS1_U  VSGJS1_L | TTCTGCTTCTTTGCCCTTGT  AAAATGAAGCGGAAATGGTG |
| VSGVO2 | VSGVO2 short_U  VSGVO1_L | ACAGAATCGGCCACAGAAAG  CATTTCCGCGTTGTCTTGTA |
| 18S | 18S_U  18S_L | GACGTAATCTGCCGCCAAAAT  AACGCCATGGCAGTCCAGTAC |
| rDNA pro | rDNAprom_U  rDNApro_L | GTCAATACAACACACAATAGG  CTTAACTGAGGAAGTGTCATA |
| rDNA sp | rDNAspacer_U  rDNAspacer_L | ATTTTCTCTACCCCTCTCTT  ATCATCGTATCATTTTCATC |
| EP cds | EP3-2U  EP3-2L | ATGGCACCTCGTTCCCTTTA  AGAATGCGGCAACGAGACCAA |
| EP pro | ProcyProm_U  ProcyProm_L | AGTTTAAGATGTTCTCGTGAT  CTTTTTGGTGTAATTGAAGTC |
| FLuc | Luc_U  Luc_L | GTGTTGGGCGCGTTATTTAT  CATCGACTGAAATCCCTGGT |
| RLuc | Ren_U  Ren_L | GATAACTGGTCCGCAGTGGT  ACCAGATTTGCCTGATTTGC |
| SL | SL_U  SL_L | CCGACACGTTTCTGGCACGACAG  TGCGTGTGTTGGCCCAGCTGCTAC |
| Myosin B | MyoB_U  MyoB_L | CTGCAGAACAAGCACGGCATT  ACGCTCAACAGTGGCAGTGAA |
| Tubulin | Tub_U  Tub_L | AGGCAACGGGAGGTCGCTATG  GGGATGGGATGATGGAGAAAG |
| U2 | U2_U  U2_L | CTGCGTGATCTTTTGTTCCT  CGACTGCGTTTTCGTATTTT |
| 5S | 5Snew_U  5Snew_L | GACCATACTTGGCCGAATG  TACAACACCCCGGGTTCC |
| a | rDNA_3303 U  rDNA_3499L | TTACGAACGTGAAGTGTAGAT  CATTTGGAACACGGTAACTAT |
| b | RibS1_U  RibS1_L | TCAGGTAGGTTGACGCAGTG  CGCGACACTCCTAAGTCTCC |
| c | rDNA sp2U  rDNA sp2L | CCAGTGGGCTGGAATTAGAA  AGGTGTATCCATTGCCGTTC |
| d | RibS2_U  RibS2_L | TCCTCGGGAAGTGATTCAAC  CCTTATCAAGCACCACACGA |
| e | NS2_U  NS2_L | ACAACCAACGACTGCTACCC  GTCAAAAGCATCGGCTCTTC |
| +30 | rDNA_30 U  rDNApro_L | GACCGATGGTGACGACATAA  CTTAACTGAGGAAGTGTCATA |
| +705 | rDNA_705 U  rDNA_890 L | ATTGCCGCTGCTTTTTACAC  TATCAGGTGCCAAGCCCTAC |
