# Supplementary material for: SUMOylation by the E3 Ligase TbSIZ1/PIAS1 Positively Regulates VSG Expression in Trypanosoma brucei
Source: PLoS Pathog. 2014 Dec 4;10(12):e1004545. doi: 10.1371/journal.ppat.1004545 (PMC4256477; doi:10.1371/journal.ppat.1004545)
Supplement: Text S1 — Supplementary materials and methods. Detailed description of Trypanosomes cell lines, Yeast two-hybrid (Y2H), 3D Microscopy, Chromatin, Immunoprecipitation (ChIP), Relative quantification by qPCR, FACS analysis, Cell extracts and Immunoblots, Immunoprecipitation, Quantitative Western Blots and Proximity Ligation Assay (PLA). (DOCX) [file ppat.1004545.s011.docx]

**Supporting Information**

**Text S1**

**Supplemental Material and Methods**

**SUMOylation of chromatin-associated proteins by TbSIZ1/PIAS1 positively regulates *VSG* expression in Trypanosomes**

**Diana López-Farfán, Jean-Mathieu Bart, Domingo I. Rojas-Barros and Miguel Navarro**

Trypanosomes cell lines

*T. brucei* bloodstream form cells (strain Lister 427, antigenic type MiTat 1.2, clone 221a) were cultured at 37°C in HMI-9 medium. Procyclic- form cells 427, were grown at 28 ºC in SDM79 supplemented with 10% (v/v) fetal calf serum. DNA transfections, and selection procedures were described previously [[1](#_ENREF_1)].

Establishment of the dual-reporter cell line, named SALR, in the ‘single-marker’ cell line [[1](#_ENREF_1)] have been described previously [[2](#_ENREF_2)]. The SALR dual reporter cell line contains a Firefly Luciferase (*FLuc*)-reporter plasmid (pMig104) integrated 405 bp downstream of the active ES promoter, and pMig113 (*Renilla*-Luciferase reporter), integrated in the tubulin locus. The generation of the SILR cell line was similar to SALR cell line, but the Firefly Luciferase (*Fluc)* containing plasmid pMig104 [[3](#_ENREF_3)], was integrated 405 pb downstream of an inactive ES promoter. pMig104 contains as targeting sequences a region located downstream of the promoter, a highly conserved sequence among different *VSG*-ESs. Reporter activities analyses allow us to identify a clone that showed low reporter activities, in contrast to the SALR reporter activities where the *FLuc* gene is inserted in the active *VSG*-ES promoter [[2](#_ENREF_2)]. We identified the site of insertion by PCR and sequencing of the flanking *FLuc* regions from SALR and SILR genomic DNAs using a primer specific for the *FLuc* gene (LucATG_L) and a primer annealing upstream of the VSG-ES promoters (ESPM2_U) (Table S1). Sequence analysis of PCR fragments demonstrated that the *FLuc* is inserted in the active *VSG221*-ES (BES1/TAR40) in the SALR cell lane, and at the same position downstream of the *VSG*-ES promoter of the BES5/TAR98 VSG800/427-18 in the SILR cell lane [[4](#_ENREF_4)].

Yeast two-hybrid (Y2H)

Bait cloning and Y2H screening were performed by Hybrigenics Services SAS, France (http://hybrigenics.com/services). The coding sequence for full-length TbRPB7 (tb11G1415, Tb11.01.6090) was cloned into a plasmid pB27 as a C-terminal fusion to LexA (N-Lex-TbRPB7). The construct was used as a bait to screen at saturation a highly complex Treu927 genomic fragment library of *T. brucei* constructed into pP6. pB27 and pP6 derive from the original pBTM116 and pGADGH plasmids, respectively. 85 million clones (11-fold the complexity of the library) were screened using a mating approach with Y187 (matα) and L40٨Gal4 (mata) yeast strains as previously described [[5](#_ENREF_5)]. 66 His+ colonies were selected on a medium lacking tryptophan, leucine and histidine. The prey fragments of the positive clones were amplified by PCR and sequenced. The resulting sequences were used to identify the corresponding interacting proteins in the *Trypanosoma brucei* Treu927 genome sequence using a fully automated procedure in the GenBank database (NCBI). A confidence score (PBS, for Predicted biological score) was attributed to each interaction as previously described [[6](#_ENREF_6)]. This global score represents the probability of an interaction being nonspecific. The PBS scores have been used to select out of the 66 clones those with high confidence interaction (Score B); including TbSIZ1, a Topoisomerase, an Ubiquitin hydrolase and a nucleoporin.

3D Microscopy

Three-dimensional immunofluorescence (3D-IF) was carried out on cells in suspension as previously described [[7](#_ENREF_7)]. Briefly, cells in PBS were fixed for 2.5 hours on ice with 4% paraformaldehyde and permeabilized with 1% NP-40 for 1 hour at room temperature. Antibodies were diluted in 1% of inactivated Normal Goat Serum in PBS (Sigma-Aldrich) as follow: mouse anti-TbSUMO mAb 1:800, mouse anti-TbSIZ1 mAb 1:10, rabbit anti-TbRPA1 affinity-purified antiserum 1:600, rabbit anti-VSG221 antiserum 1:3000, rabbit anti-GFP antiserum 1:5000 (Invitrogen), and mouse anti-GFP mAb, 1:600 (Invitrogen). Alexa Fluor 488 and Alexa Fluor 594 goat anti-mouse or anti-rabbit (Invitrogen) were used as secondary antibodies. Cells were DAPI stained. Stacks (0.2-μm z step) acquisition was performed with a microscope system (Cell R IX81; Olympus), 100x objective, NA 1.40, illumination system (MT20; Olympus), and camera (Orca CCD; Hamamatsu). Deconvolution of 3D images was performed using Huygens Essential software (version 2.9; Scientific Volume Imaging) using an experimentally calculated point-spread function with 0.2-μm TetraSpeck microspheres (Invitrogen). Pseudocoloring, colocalization analysis and maximum intensity projections were performed using ImageJ program version 1.43 software (National Institutes of Health). For the colocalization mask, we used the plugin “Colocalization_.class”, where two points are considered as colocalizated if their respective intensities are strictly higher than the threshold of their channels, which we set up at 80% and if their ratio of intensity is higher than the ratio setting value of 80%.

Chromatin Immunoprecipitation (ChIP)

*T. brucei* bloodstream form cultures were fixed by addition of 37% formaldehyde solution (Sigma-Aldrich) to a final concentration of 1% and incubated at 37º for 15 min. To stop the cross-linking reaction 2.5 M glycine was added to a final concentration of 125 mM. Cells were collected by centrifugation at 3500 rpm for 20 min at 4ºC, washed in cold PBS and centrifuged again. Pellet was resuspended in 10 ml of PBS with protease inhibitor cocktail (Roche), centrifuged at 4000 rpm for 15 min, at 4ºC and stored at -80ºC. Pellet was thawed and cells were resuspended in 1ml of lysis buffer per 10^8^ cells (1% SDS; 10mM EDTA pH 8.0; 50 mM TrisHCl pH 8.0; 1mM PMSF; 1mM DTT and protease inhibitor cocktail) and incubated on ice 10 min. Cells were sonicated in a VibraCell sonicator to shear the chromatin to ~300pb in length (9 cycles of 30 sec on/30 sec off) and centrifuged at 13000 rpm for 5 min at 4°C, the supernatant containing sheared chromatin was frozen at -80C. Chromatin was thawed on ice, diluted 1:5 in ChIP dilution buffer (0.01%SDS; 1.1% Triton X-100; 1.2 mM EDTA, 16.7 mM TrisHCl pH 8.0; 150 mM NaCl; 1mM PMSF; 1mM DTT and protease inhibitor cocktail) and pre-cleared with Sepharose 4B beads (Sigma-Aldrich) (blocked previously in TE buffer with 0.2 mg/ml salmon sperm DNA and 1 mg/ml BSA) for 1hr at 4ºC. An aliquot of the input DNA (10%) was saved. 2.5 ml of pre-cleared chromatin (5 x 10^7^ cells per ChIP) was incubated overnight at 4ºC with each antibody (6 μg of anti-TbRPA1 affinity-purified antiserum, 60 μg of anti-TbSUMO 1C9H8, 60 μg of unspecific antiserum). Next, 40 µl of 50% slurry of blocked protein G Sepharose fast flow (Sigma-Aldrich) was added and incubated for 1hr at 4ºC; immune complexes were collected by centrifugation at 1000 g for 2 min and washed for 5 min with 1 ml of each one the following buffers: low salt wash buffer (0.1%SDS; 1% Triton X-100; 2 mM EDTA pH 8.0; 20 mM TrisHCl pH 8.0; 150 mM NaCl), high salt wash buffer (0.1%SDS; 1% Triton X-100; 2 mM EDTA pH 8.0; 20 mM TrisHCl pH 8.0; 500 mM NaCl), LiCl buffer (0.25 M LiCl; 1% NP-40; 1% Na-Deoxycholate; 1 mM EDTA pH 8.0; 10 mM TrisHCl pH 8.0), and twice with TE buffer (10 mM TrisHCl pH 8.0; 1 mM EDTA pH 8.0). The complexes were eluted from the beads with 500 μl of elution buffer (1%SDS, 0.1M NaHCO3) by incubation for 30min at RT with rocking. Crosslinks were reversed by incubating at 65°C for 16h with 20 μl 5M NaCl (200mM final concentration). RNase A was added to a final concentration of 100 μg/ml and incubated for 1hr at 37ºC. Then 10 μl of 0.5 M EDTA, 20 μl of 1M Tris-HCl, pH 6.5 and 8 μl of 20 mg/ml Proteinase K (200 μg/ml final concentration) were added and incubated for 2 hr at 55ºC. Samples were extracted with phenol-chloroform 1:1 and ethanol precipitated. DNA was resuspended in 50 μl of H_2_O and assayed by quantitative PCR (qPCR). qPCR was performed using the SYBR green supermix (Quanta Biosciences) in a CFX96 cycler (BioRad), qPCR mixtures contained 2µl of a 1:5 dilution of the ChIPed DNA or a 1:50, 1:100, 1:200 dilution of the input sample and 500nM of each primer. All reactions were performed in duplicate and each product was verified by melting curve analysis. Standard curves for each primer pair were generated with serial dilutions of input DNA to determine PCR efficiency. Immunoprecipitation values were normalized to the amount of input chromatin using the standard curve equation we obtained the relative amount of each specific fragment in the ChIPed DNA and in the input DNA, then we calculated the percentage of input immunoprecipitated. Finally, the background values obtained with a non-specific ChIP control (pre-bleed antiserum) were subtracted from the values obtained with the specific antibodies. Independent ChIP experiments were performed at least three times and statistical analysis (Student´s t-test) was applied to compare data sets.

Relative quantification by qPCR

Relative quantification of PCR fragments showed in Figure S5A was performed by qPCR as described before with genomic DNA template and the quantification was performed using the △△CT method and *FLuc* single copy gene was used as the reference sample.

FACS analysis

For cell cycle analysis, samples (1.5 x 10^7^ cells) were collected, centrifuged (1400 xg at 4ºC for 10 min), washed in 5ml 1X PBS and centrifuged again. Pellets were resuspended in 70% ethanol - 30% 1X PBS solution and incubated 1hr at -20ºC. After incubation the cells were centrifuged and washed in 5ml 1X PBS and resuspended in 500µl 1X PBS. Propidium iodide (PI) and RNase were added to 40 µ/ml and 10 µ/ml final concentration, respectively, and incubated 30min at RT. DNA content of 30,000 cells per sample was analysed with a FACScan flow cytometer using the Cellquest software (BD Biosciences). Gating was determined with control cells and the same values were used for all treated cells.

Cell extracts and Immunoblots

Parasites from 20 ml cultures at density of ~1.5 x 10^6^ cells per ml were collected by centrifugation and washed once in Trypanosome Dilution Buffer (TDB) with 1X protease inhibitor cocktail (Roche) and 0-40mM N-ethylmaleimide (NEM). Pellets were resuspended in the same buffer at concentration of 1 x 10^6^ cells per µl. Whole cell lysates were prepared by diluting 1:1 in 2x Laemmli sample buffer and heated at 95ºC for 5 min. Samples were loaded (~5 x 10^6^ cells per lane) in 4–20% precast polyacrylamide gels (Bio-Rad Laboratories) and analysed by Western blot using anti-TbSUMO mAb ascites (1:500), anti-TbSIZ1 mAb ascites (1:1000), anti-UBC9 mouse antiserum (1:4000), anti-Tubulin (1:5000) or anti-TOR4 polyclonal antibody (1:1000) [[8](#_ENREF_8)].

Immunoprecipitation

For each Immunoprecipitation (IP) experiment 3.3 x 10^9^ Bloodstream form (BF) cells were used. BF parasites used in IP experiments carried out upon TbSIZ1 KD were collected from 48h-induced cultures. Cells were washed in TDB with 1X protease inhibitor cocktail (Roche) and 20mM NEM. Pellets were resuspended at ~3.0 x 10^9^ cells ml^-1^ in hypotonic buffer (25 mM Hepes pH 7.5, 5mM KCl, 0.5 mM MgCl2, 20 mM NEM, 2X protease inhibitor cocktail (Roche)) and centrifuged at 3.500 g for 10 min at 4ºC. The pellet was resuspended in 5 ml of hypotonic buffer, then 5 ml of hypotonic buffer with 1.2% NP40 was added and the extract was incubated for 5 min at 4ºC and centrifuged again, this step was performed twice but the second time the extract was centrifuged at 16.000 rpm for 30 min at 4ºC. This pellet was resuspended at 5.0 x 10^9^ cells ml^-1^ in urea buffer (6M Urea, 50mM Hepes pH 7.5, 500 mM NaCl, 20mM NEM, 0.5% NP40, 2x protease inhibitor cocktail (Roche)) and sonicated until losing the viscosity (approx. 15 min). The cell lysate was centrifuged at 16.000 rpm for 30 min at RT and the supernatant with the nuclear enriched fraction was stored at -80 ºC. For immunoprecipitations, the nuclear extract was diluted 1:6 with dilution buffer (50 mM Hepes pH 7.5, 80 mM NaCl, 0.5% NP40, 0.1% SDS, 0.1mM EDTA, 10 mM NEM, 1X protease inhibitor cocktail (Roche)) followed by centrifugation at 14.800 rpm for 10 min at 4ºC to remove any precipitated. Protein G Sepharose (Sigma-Aldrich) incubated previously with antibodies was added to each 4 ml of diluted extract obtained form 3.3 x 10^9^ cells and incubated overnight at 4ºC. Antibodies concentration for IP experiments was 200 µg ml^-1^ of anti-TbSUMO mAb 1C9H8, 200 µg ml^-1^ of anti-TOR4 mAb 5H7 [[8](#_ENREF_8)], 1.5 µg ml^-1^ of anti-TbRPA1, and 75 µg ml^-1^ of unspecific IgGs (pre-bleed antiserum). Beads were washed five times for 10min at 4ºC on a rotating wheel with 1ml of wash buffer (50 mM Hepes pH 7.5, Urea 1M, 150 mM NaCl, 0.5% NP40, 0.1% SDS, 0.1mM EDTA, 10 mM NEM, 1X protease inhibitor cocktail (Roche)), then washed once with 1ml of high-salt buffer (wash buffer with 500 mM NaCl instead of 150 mM) for 5 min at RT. A final wash with PBS 1X was carried out for 5 min. Beads were eluted with 2x Laemmli sample buffer, boiled at 95ºC for 5 min. IP samples and inputs were subjected to SDS-PAGE and western blotting using the appropriated antibodies. (See above, Cell extracts and immunoblots).

Quantitative Western Blots

For quantitative Western blots total protein extracts from 5x10^5^ parasites were loaded (with the exception of the parental dilution series) on a 10% acrylamide Bis-Tris gel. After electrophoresis proteins were transferred to a nitrocellulose membrane. Membrane was incubated with blocking buffer (PBS 1X 0.05% tween (PBS-T), 5% non-fat milk) prior to incubation with rabbit polyclonal antibodies against VSG-221 (1:50.000) and BIP (1:3.000) 1 hour at RT, after washing with PBS-T, secondary antibody goat anti-rabbit IgG (H+L) 800 Dylight was applied for 90 minutes at RT (1:1.000, Thermo-Fisher). Membrane was scanned using an LI-COR Odyssey scanner and analyzed using Odyssey IR imaging software 3.0.42. Scan settings were medium image quality, 169 μm resolution and intensity 2.5–5.0 for both channels. Antibody signals were analyzed as integrated intensities of regions defined around the blots of interest in either channel. A standard curve based on BiP-normalized anti- VSG221 signal intensity was generated using different concentrations of parental cell extracts (R2 = 0.99). The standard curve regression was used to determine VSG221 expression levels in TbSUMO-depleted cell lines.

Proximity Ligation Assay (PLA)

The PLA assay was done according to the manufacturer’s protocol (Olink Bioscience, Uppsala, Sweden). Cells were dried on slides, fixed 20 min in 4% PFA and permeabilized 20 min with 1% NP40. After blocking, primary antibodies (mouse anti-TbSUMO 1C9H8 (1:2000) and rabbit anti-TbRPA1 affinity-purified polyclonal (1:600)) were incubated 1h. Then, the PLA probe solution containing the secondary antibodies conjugated with oligonucleotides (PLA probe MINUS and PLUS) were applied. Secondary species-specific antibodies conjugated with oligonucleotides would hybridize to the two PLA probes if they were in close proximity (<40nm). The slides were then incubated with the ligation solution together with the ligase which would join the two hybridized oligonucleotides to a closed circle. The amplification mix containing nucleotides and polymerase was applied to the slides. The oligonucleotide arm of one of the PLA probes acts as a primer for a rolling circle amplification (RCA) reaction, generating a repeated sequence and extended product. Finally, the nuclei DNA were stained with DAPI and the slides were examined under fluorescent microscopy. As negative control and to discard cross-reaction signal, complete protocol was performed in parallel using primary antibodies separately.

**Supplemental References**

1. Wirtz E, Leal S, Ochatt C, Cross GA (1999) A tightly regulated inducible expression system for conditional gene knock-outs and dominant-negative genetics in Trypanosoma brucei. Mol Biochem Parasitol 99: 89-101.

2. Penate X, Lopez-Farfan D, Landeira D, Wentland A, Vidal I, et al. (2009) RNA pol II subunit RPB7 is required for RNA pol I-mediated transcription in Trypanosoma brucei. EMBO Rep 10: 252-257.

3. Navarro M, Cross GA (1998) In situ analysis of a variant surface glycoprotein expression-site promoter region in Trypanosoma brucei. Mol Biochem Parasitol 94: 53-66.

4. Hertz-Fowler C, Figueiredo LM, Quail MA, Becker M, Jackson A, et al. (2008) Telomeric expression sites are highly conserved in Trypanosoma brucei. PLoS ONE 3: e3527.

5. Fromont-Racine M, Rain JC, Legrain P (1997) Toward a functional analysis of the yeast genome through exhaustive two-hybrid screens. Nat Genet 16: 277-282.

6. Formstecher E, Aresta S, Collura V, Hamburger A, Meil A, et al. (2005) Protein interaction mapping: a Drosophila case study. Genome Res 15: 376-384.

7. Landeira D, Bart JM, Van Tyne D, Navarro M (2009) Cohesin regulates VSG monoallelic expression in trypanosomes. J Cell Biol 186: 243-254.

8. Barquilla A, Saldivia M, Diaz R, Bart JM, Vidal I, et al. (2012) Third target of rapamycin complex negatively regulates development of quiescence in Trypanosoma brucei. Proc Natl Acad Sci U S A 109: 14399-14404.
